# Prediction of 30-day mortality in heart failure patients with hypoxic hepatitis: Development and external validation of an interpretable machine learning model

**DOI:** 10.3389/fcvm.2022.1035675

**Published:** 2022-10-28

**Authors:** Run Sun, Xue Wang, Haiyan Jiang, Yan Yan, Yansong Dong, Wenxiao Yan, Xinye Luo, Hua Miu, Lei Qi, Zhongwei Huang

**Affiliations:** ^1^Department of Emergency Medicine, Affiliated Hospital of Nantong University, Nantong, China; ^2^Medical School of Nantong University, Nantong University, Nantong, China; ^3^Health Management Center, Affiliated Hospital of Nantong University, Nantong, China

**Keywords:** hypoxic hepatitis, heart failure, machine learning, interpretability, prediction model

## Abstract

**Background:**

This study aimed to explore the impact of hypoxic hepatitis (HH) on survival in heart failure (HF) patients and to develop an effective machine learning model to predict 30-day mortality risk in HF patients with HH.

**Methods:**

In the Medical Information Mart for Intensive Care (MIMIC)-III and IV databases, clinical data and survival situations of HF patients admitted to the intensive care unit (ICU) were retrospectively collected. Propensity Score Matching (PSM) analysis was used to balance baseline differences between HF patients with and without HH. Kaplan Meier analysis and multivariate Cox analysis were used to determining the effect of HH on the survival of CF patients. For developing a model that can predict 30-day mortality in CF patients with HH, the feature recurrence elimination (RFE) method was applied to feature selection, and seven machine learning algorithms were employed to model construction. After training and hyper-parameter optimization (HPO) of the model through cross-validation in the training set, a performance comparison was performed through internal and external validation. To interpret the optimal model, Shapley Additive Explanations (SHAP) were used along with the Local Interpretable Model-agnostic Explanations (LIME) and the Partial Dependence Plot (PDP) techniques.

**Results:**

The incidence of HH was 6.5% in HF patients in the MIMIC cohort. HF patients with HH had a 30-day mortality rate of 33% and a 1-year mortality rate of 51%, and HH was an independent risk factor for increased short-term and long-term mortality risk in HF patients. After RFE, 21 key features (21/56) were selected to build the model. Internal validation and external validation suggested that Categorical Boosting (Catboost) had a higher discriminatory capability than the other models (internal validation: AUC, 0.832; 95% CI, 0.819–0.845; external validation: AUC, 0.757 95% CI, 0.739–0.776), and the simplified Catboost model (S-Catboost) also had good performance in both internal validation and external validation (internal validation: AUC, 0.801; 95% CI, 0.787–0.813; external validation: AUC, 0.729, 95% CI, 0.711–0.745).

**Conclusion:**

HH was associated with increased mortality in HF patients. Machine learning methods had good performance in identifying the 30-day mortality risk of HF with HH. With interpretability techniques, the transparency of machine learning models has been enhanced to facilitate user understanding of the prediction results.

## Introduction

Heart failure (HF) is a serious end-stage cardiac event where hyperemia or hypoperfusion associated with reduced cardiac output and cardiac dysfunction significantly lead to other organ damage (1). Liver disease is common in patients with HF, as it is highly sensitive to changes in blood flow. Approximately 20–30% of HF patients develop liver dysfunction as a result of impaired cardiac function (2, 3). Hepatic congestion and/or impaired arterial perfusion may contribute to liver damage in the context of HF, including liver congestion, cardiac cirrhosis, and, most severely, hypoxic hepatitis (HH) (3).

HH is a specific acute liver injury, also known as “hypoxic liver injury,” “shock liver,” etc. and one of its pathological features is a massive and transient increase in serum transaminase activity resulting from hypoxic necrosis of hepatocytes in centrilobular regions (4–6). In the intensive care unit (ICU), HH is not uncommon; while, HF, respiratory failure, and septic shock are the disease basis in over 90% of cases (5). HH was previously thought to be a hypoxic event caused by rapid changes in hepatic blood flow. Henrion et al. determined that the underlying mechanisms of HH in different disease backgrounds may vary considerably by continuously monitoring the hemodynamics of HH patients. Patients with circulatory failure-related HH often experience a shocking state. However, no hypotension or shock state episodes have been observed in at least 50% of patients with chronic HF -related HH (4). A similar phenomenon was also observed in the studies conducted in Ebert EC and Tapper EB (7, 8).

About 49.1–94% of HH occurrences have the disease basis of HF (9–11). HF can result in a long period of passive congestion in the liver, impaired hepatic regulation of blood flow, and minor hemodynamic disorders that can trigger hypoxic necrosis of the liver. Hypoxia may also lead to liver injury when chronic circulatory stress is present, when a reduced cardiac function does not guarantee the proper perfusion and metabolism of the liver, and when the hepatocytes are unable to compensate for oxygen demand (8, 12, 13). A large retrospective study found that HH is not uncommon in patients suffering from HF and is found in approximately 5.4% of these patients (14). A more serious challenge is the ultra-high mortality rate of HF patients with HH, which can be as high as 24–40% (11, 15). There are currently several studies on HH that include not only the entire population of HH patients in the ICU but also studies based on specific disease backgrounds, such as coronavirus disease 2019 (COVID-19), cardiogenic shock, and cardiac arrest, among others (16–19). Though HF is the most common disease basis for HH, only a few studies have been performed on patients with HF-based HH. For this reason, we focused on HF patients with HH in this study to understand the impact of HH on the short- and long-term survival of these patients. As there are no non-invasive and convenient tools to assess the prognosis of these patients, we developed machine learning models based on early clinical data to evaluate the mortality risk. To enhance model transparency and reveal the effects of relevant features on the short-term survival of patients, multiple machine learning interpretation techniques were used, including the SHapley Additive Explanations (SHAP) (20, 21), the Local Interpretable Model-Agnostic Explanations (LIME) (22), and the Partial Dependence Plot (PDP) (23).

## Materials and methods

### Data source

The data for this retrospective study was obtained from the Intensive Care Medicine Information Mart (MIMIC) database. Clinical information of chronic HF patients admitted to ICU was extracted from MIMIC-III (version 1.4) and MIMIC-IV (version 2.0) databases (24, 25). The public databases were provided by the Massachusetts Institute of Technology’s Computational Physiology Laboratory (MIT, Cambridge, Massachusetts, USA) (26), and include clinical information on patients admitted to the ICU at Beth Israel Deaconess Medical Center (BIDMC, Boston, MA, USA). The EICU Collaborative Database (EICU CRD) (version 2.0) contains patient information from numerous ICUs for external validation of predictive models (27, 28). According to the Health Insurance Portability and Accountability Act (HIPAA), all patients participating in this program were de-identified. The current project had no impact on clinical care and therefore was exempt from the requirement to obtain individual consent. The team members Run Sun, who had access to the above databases, were responsible for data extraction after signing the PhysioNet Credentialed Health Data Use Agreement (cite number: 45997657).

### Patient

This study examined adult patients with chronic HF who were admitted to the ICU for more than 24 h, while patients with multiple ICU admissions were analyzed using the first record.

Patients with other disorders potentially causing elevated transaminase levels were excluded from the study (11, 29, 30): (1) viral hepatitis; (2) liver failure or liver necrosis; (3) cirrhosis and chronic liver diseases; (4) toxic hepatitis; (5) liver injury; (6) Hepatic infarction; (7) autoimmune hepatitis; (8) liver and near liver surgery; (9) Other conditions associated with abnormal liver function tests, such as cholangitis and pancreatitis; (10) Rhabdomyolysis (Supplementary Table 1).

Hypoxic hepatitis: There are currently no definitive diagnostic criteria for HH. We referred to the diagnostic criteria in the largest study of HH to date, which are based on the presence of circulatory impairment and transaminases [alanine transaminase (ALT)/aspartate transaminase (AST)] exceeding five times the upper limit of normal after ruling out other potential causes of liver function abnormalities (11). In histological studies, HH has been shown to occur not only in patients with extremely elevated transaminases but also in those with moderately elevated transaminase levels (31). Notably, all of the elevated transaminase levels in the HH patients included in this study occurred during their ICU stay.

### Data extraction and management

Based on structured language (SQL), the following data were collected by Navicat premium version 15.0.12 (premium soft Cybertech Ltd., Hongkong): (1) Length of hospital stay, ICU stay, 30, 90, 180, 365 days survival status (first day of ICU admission as day 0); (2) Demographic information: age, gender; (3) Chronic comorbidities Hypertension, Dyslipidemia, Diabetes, Coronary surgery history, Old myocardial infarction, Cardiomyopathy, Atrial fibrillation, Chronic pulmonary disease, Chronic kidney disease, Peripheral vascular diseases, Cerebrovascular diseases, Hypothyroidism and Cancer; (4) Vital signs and urine output within 24 h of the ICU stay, where vital signs are recorded as the mean value; (5) First laboratory test results within 24 h of ICU admission; (6) Treatment within 24 h of ICU admission: mechanical ventilation (MV), renal replacement therapy (RRT), vasoactive agents including dopamine, epinephrine, norepinephrine and phenylephrine, blood product transfusion including red blood cell (RBC) and fresh frozen plasma (FFP); (7) simplified acute physiology score II (SAPS II), sequential organ failure (SOFA) score within 24 h of ICU admission.

A continuous variable that exhibited missing rates exceeding 30% was excluded, and the remaining missing data were subjected to multiple imputations by using the “mice” package in R (32). Detailed information regarding missing rates, processing methods, and data distribution before and after imputation can be found in Supplementary Tables 2-1–2-3.

### Data analysis

The Shapiro-Wilk test was performed to determine whether or not the samples conformed to a normal distribution. Continuous variables meeting a normal distribution were expressed as mean + standard deviation (SD), non-normal continuous variables as median (interquartile range, IQR), and categorical variables as frequencies and percentages, depending on the distribution. Data with non-normal distributions or unequal variances were analyzed with non-parametric tests (Mann-Whitney-*U*-test), and categorical variables were analyzed with Pearson chi-square tests. Propensity Score Matching (PSM) was employed to balance 53 baseline characteristics of HF patients with and without HH. To study the impact of HH on the mortality of HF patients, Kaplan Meier survival curves and Log-rank tests were applied to compare the mortality rates of the two groups at 30, 90, 180, and 365 days, respectively. Multivariate Cox regression analysis was undertaken to investigate the effect of HH on the mortality rate of HF patients.

### Variable selection and model development

The predictive goal of the model was the 30-day mortality in HF patients with HH. Generally, constructing a model based on valuable variables can result in better accuracy, but too many variables can cause a “dimension disaster,” which reduces model accuracy and applicability. Feature recursive elimination- cross-validation (RFE-CV) was performed based on XGBoost (eXtreme Gradient Boosting) to eliminate redundant features. This method specifies a machine learning algorithm that obtains the optimal number of features by computing the validation scores for each subset and choosing the features with the highest validation score. SAPS II and SOFA scores are widely used in clinical practice to assess patient prognosis. In models that included the disease score as a feature, the score plays a key role in ensuring prediction accuracy. In actual clinical practice, obtaining an accurate disease score depends on the completeness of the information constituting the score; thus, incorporating the score into model construction may reduce its usefulness. In this study, disease scores were not used for variable screening and model construction but rather for comparison with the optimal machine learning model.

HF Patients with HH from the MIMIC database were randomly divided into training and testing sets by a 7:3 ratio, whereas the EICU dataset was designated for external validation. The ratio of the survivor group to the non-survivor group is about 2: 1, which is unbalanced, and in this case, the model’s prediction results may be biased toward a more class of events, leading to high precision but low recall (sensitivity). The training set data were resampled using the STOME resampling technique to address the data imbalance. Models were constructed using seven machine learning methods, including classical logistics regression (LR), decision tree (DT), and support vector machine (SVM), along with the integrated learning models random forest (RF), categorical boosting (CatBoost), extreme gradient boosting (XGBoost), and light gradient boosting machine (LightGBM). Each model underwent hyperparameter optimization (HPO) and 10-fold cross-validation on the training set, followed by comparisons with an independent testing set and an external validation cohort. HPO was performed based on the open source optimization framework **Optuna** (version 2.10.0) (33). After determining an approximate search interval for the hyperparameters based on the learning curve (i.e., an interval within which there was no obvious overfitting and model overfitting), HPO was performed to obtain the best combination of model hyperparameters. Each model was trained 300 times during the HPO process. The main index of the performance evaluation was the area under the receiver operating characteristic curve (AUC), and the secondary indexes were Matthews correlation coefficient (MCC) score (34), F1 score, accuracy, and recall. In addition, the calibration was plotted to evaluate the consistency between the model’s predicted probability and the actual probability, and the Brier score was used to assess the model’s calibration. A combination of SHAP, LIME, and PDP was used to interpret the model at the global and local levels to avoid the contingency caused by a single model interpretation method. In this study, all statistical analyses were performed using Python 3.9.0 (Python Software Foundation) and R software 4.0.4 (R Foundation for Statistical Computing, Vienna, Austria). Two-tailed tests were performed, and *P*-values < 0.05 were considered statistically significant. Supplementary Figure 1 is a flow diagram of the overall study design.

## Result

### Characteristics of patients

The patient screening procedure is described in Supplementary Figure 1. In the MIMIC cohort, 17,214 patients with chronic HF were included, 1,114 of whom presented with HH, representing an incidence of 6.5%. The EICU cohort included 6,923 patients with chronic HF, and 383 developed HH, representing a 5.5% incidence rate.

The baseline data for non-HH and HH patients are given in Supplementary Table 3. Before PSM, the HH group was older and had a higher proportion of male patients. The HH group also has a higher proportion of patients with chronic lung disease, cardiomyopathy, and cerebrovascular disease, as well as a lower proportion of hypertensive patients. Patients in the HH group obtained a higher disease score on their first day of ICU admission, as well as higher heart and respiratory rates, lower blood pressure levels, and lower 24-h urine output. Laboratory tests showed that patients in the HH group had more severe anemias, electrolyte imbalances, and impaired coagulation functions. Furthermore, a greater percentage of patients in the HH group received FFP infusions, vasoactive drug support, RRT, and MV. After PSM, 1,096 patients were included in each of the two groups, and the standardized mean difference (SMD) between the groups was significantly lower than before (Supplementary Figure 2). Except for the white blood cell count and glucose (*P* < 0.05), there were no significant differences in the characteristics between the two groups (Supplementary Table 3).

Table 1 depicts the characteristics selected based on FRE between survivor and non-survivor groups of HF patients with HH. As shown in the MIMIC cohort, patients in the non-survivor group were older, had lower systolic blood pressure, and greater severity of anemia and infection. The non-survivor group also had worse renal function, as reflected by higher blood urea nitrogen (BUN), serum creatinine (SCR), and less urine output. Further, the non-survivor group had a higher anion gap and lactate value, indicating more severe acidosis. Regarding therapy, a higher proportion of patients in the non-survivor group received MV and vasoactive drugs. Except that there were no statistical differences between survivors and non-survivors in hemoglobin and lactate, the EICU cohort followed similar trends to the MIMIC cohort. Surprisingly, ALT levels were lower in the non-survivor group than in the survivor group, both in the MIMIC and EICU cohorts. The full characteristics of HF patients with HH are presented in Supplementary Table 4.

**TABLE 1:** Characteristics between survivor and non-survivor groups of heart failure patients with hypoxic hepatitis in MIMIC and EICU database.

	MIMIC database			EICU database		
		
Variables	Survivor (*n* = 736)	Non-survivor (*n* = 378)	*P*-value	Survivor (*n* = 225)	Non-survivor (*n* = 82)	*P*-value
Age	71 (60.79)	77 (68.84)	<0.001	68 (57.76)	73 (63.81)	0.001
Gender (female), %	293 (39.81)	151 (39.95)	0.965	83 (36.89)	37 (45.12)	0.191
Atrial fibrillation, %	346 (47.01)	190 (50.26)	0.303	60 (26.67)	19 (23.17)	0.535
Systolic pressure, mmHg	109 (101.119)	106 (98.115)	<0.001	105 (97.118)	103 (95.111)	0.072
SpO2, %	97 (96.98)	97 (95.99)	0.908	97 (92.98)	96 (59.98)	0.926
Urine output, ml/24 h	1690 (945, 2694)	1019 (435, 1870)	<0.001	1275 (708, 2450)	525 (208,1376)	<0.001
Hemoglobin, g/dL	11.3 (9.5, 13.0)	10.7 (9.3, 12.2)	<0.001	11.6 (9.4, 13.4)	10.7 (9.3, 13.0)	0.292
MCHC, g/dL	33.2 (32.2, 34.2)	32.6 (31.4, 33.9)	<0.001	32.9 (31.8, 33.7)	32.3 (31.4, 33.1)	0.017
Platelet, × 10^9/L	208 (156,275)	199 (140,281)	0.068	188 (142,246)	178 (136,250)	0.558
RDW, %	14.5 (13.5, 16.2)	15.3 (14.2, 16.9)	<0.001	15.6 (14.3, 17.3)	16.7 (14.9, 18.4)	0.005
WBC, × 10^9/L	12.8 (9.9, 16.6)	14.4 (9.9, 19.3)	0.005	11.8 (9.0, 15.7)	13.7 (9.7, 19.8)	0.035
Anion gap, mmol/L	16.0 (14.0, 18.0)	18.0 (15.0, 21.0)	<0.001	12.1 (10.0, 16.0)	15.0 (10.8, 19.0)	0.03
Sodium, mmol/L	138 (135,140)	138 (135,141)	0.289	137 (133,140)	138 (135,140)	0.405
BUN, mg/dL	26 (18.44)	37 (24.55)	<0.001	32 (22.53)	37 (26.59)	0.016
SCr, mg/dL	1.2 (0.9, 1.8)	1.6 (1.1, 2.6)	<0.001	1.6 (1.1, 2.5)	1.9 (1.4, 2.8)	0.011
ALT	140 (70,273)	128 (49,265)	0.017	152 (45,422)	87 (34,237)	0.044
Lactate	2.2 (1.6, 3.3)	2.9 (1.8, 5.2)	<0.001	2.5 (1.6, 4.5)	2.6 (1.7, 7.2)	0.116
RBC trans, %	151 (20.52)	94 (24.87)	0.097	11 (4.89)	3 (3.66)	0.648
Dopamine, %	123 (16.71)	96 (25.40)	0.001	14 (6.22)	4 (4.88)	0.657
Norepinephrine, %	214 (29.08)	197 (52.12)	<0.001	39 (17.33)	29 (35.37)	0.001
MV, %	351 (47.69)	253 (66.93)	<0.001	71 (31.56)	45 (54.88)	< 0.001

SpO2, saturation of pulse oxygen; MCHC, mean corpuscular hemoglobin concentration; RDW, red blood cell distribution width; WBC, white blood cell count; BUN, blood urea nitrogen; SCr, serum creatinine; ALT, alanine transaminase; RBC trans, red blood cell transfusion; MV, mechanical ventilation.

### Hypoxic hepatitis is independently associated with the mortality of patients with heart failure

**FIGURE 1:**
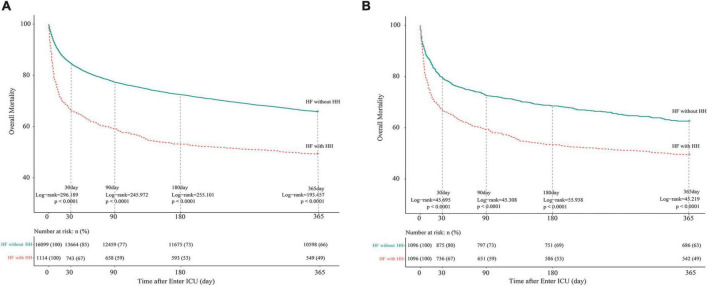
Kaplan-Meier survival curves of HF patients with and without HH. **(A)** Before PSM; **(B)** after PSM. The differences between the HF with- and without-HH groups were measured by the two-side log-rank test with a *P*-value < 0.05. HH, hypoxic hepatitis; HF, heart failure; PSM, propensity score matching.

Figures 1A,B are the Kaplan-Meier survival curves between the HH and non-HH groups before and after PSM. The all-cause mortality rates of the HH group at 30, 90, 180, and 365 days were 33, 41, 47, and 51%, respectively, and the log-rank test indicated that these rates are significantly higher in HH patients than in non-HH patients before and after the PSM (*P* < 0.0001). After adjusting for variables from demography, comorbidity, vital signs, laboratory examination, and disease score, multivariate COX regression showed that HH was an independent risk factor for increased 30-day (Before PSM: aHR = 1.722, 95% CI 1.530–1.938, *p* < 0.001;After PSM: aHR = 1.798, 95% CI 1.518–2.128, *p* < 0.001), 90-day (Before PSM: aHR = 1.565, 95% CI 1.408–1.738, *p* < 0.001; After PSM: aHR = 1.690, 95% CI 1.456–1.962, *p* < 0.001), 180-day (before PSM: aHR = 1.564, 95% CI 1.418–1.725, *p* < 0.001;After PSM: aHR = 1.709, 95% CI 1.487–1.964, *p* < 0.001), and 365-day (before PSM:aHR = 1.450, 95% CI 1.321–1.591, *p* < 0.001; After PSM: aHR = 1.570, 95% CI 1.379–1.788, *p* < 0.001) all-cause mortality in both the original cohort and PSM cohort. Detailed results of multivariate Cox regression analysis are presented in Supplementary Figures 3-1–3-4.

### Development and evaluation of models

**FIGURE 2:**
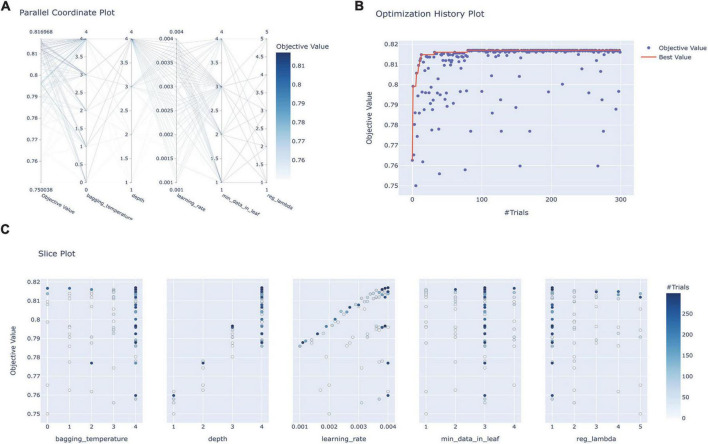
Hyperparameter optimization process for the Catboost model. **(A)** Parallel coordinate system plot of the hyperparametric distribution corresponding to different levels of AUC values, where darker colors correspond to greater AUC values; **(B)** optimization history plot illustrating the evolution of optimal values during hyperparameter optimization; **(C)** slice plot visualizing the correlation between each parameter and the AUC. CatBoost, categorical boosting.

In conducting FRE-CV on the 56 variables associated with prognosis, the accuracy scores peaked when the number of variables was 21 (Supplementary Figure 4). The feature rankings of variables in the FRE process are shown in Supplementary Table 5. Using these 21 variables, seven models were subjected to HPO in the training set, and the hyperparameter combination with the highest AUC score in cross-validation was identified after 300 trials. Figure 2 illustrates the HPO process for the Catboost model. Figure 2A shows the distributions of parameters within the Catboost model during hyperparameter optimization, with darker colors representing higher target values (AUC) levels. Figure 2B illustrates the trajectory of best value change with an increasing number of training sessions during the HPO process (Orange Line). Figure 2C shows the correlation between each parameter and AUC. The HPO process of other models is depicted in Supplementary Figures 5-1–5-6; the hyperparameter range and final set parameters are given in Supplementary Table 6; and cross-validation results are shown in Supplementary Table 7.

**FIGURE 3:**
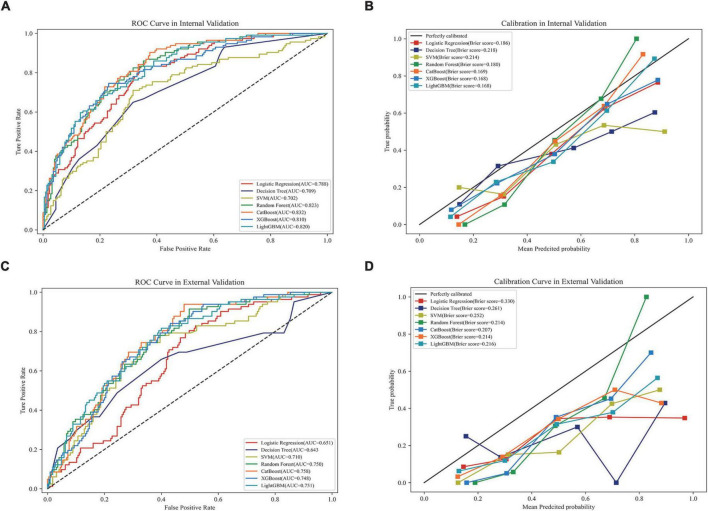
Performance of the models in internal and external validation. **(A)** ROC curves of the seven models in internal validation; **(B)** calibration curves of the seven models in internal validation; **(C)** ROC curves of the seven models in external validation; **(D)** calibration curves of the seven models in external validation. ROC, receiver operating characteristic curve; AUC, area under the curve; SVM, support vector machine; XGBoost, eXtreme gradient boosting; CatBoost, categorical boosting; LightGBM, light gradient boosting machine.

In internal validation, Catboost achieved the highest AUC score (AUC, 0.823; 95CI%, 0.819–0.845) (Figure 3A), while the calibration of the LightGBM and XGBoost models was the best (Brier score, 0.168; 95% CI, 0.162–0.174), followed by the calibration of the Catboost model (Brier score, 0.169; 95% CI, 0.162–0.174) (Figure 3B). As a result of external validation, the Catboost had the highest AUC among other models (AUC, 0.757; 95% CI, 0.739–0.776) (Figure 3C), and in terms of calibration, the Catboost outperformed other models as well (Brier Score, 0.207; 95%CI, 0.202–0.211) (Figure 3D). The overall performance of the seven models in internal and external validation can be found in Table 2, where the Catboost model outperformed others in terms of MCC score, F1 score, recall, and accuracy. The learning curve of each model in the training process with final parameters is provided in Supplementary Figure 6.

**TABLE 2:** Summary of models’ performance in internal and external validation.

Model	AUROC (95% CI)	Accuracy	Recall	F1-score	MCC	Brier-score (95% CI)
**Internal validation**						
LR	0.788 (0.774–0.801)	0.71	0.700	0.690	0.386	0.186 (0.182–0.191)
SVM	0.702 (0.677–0.721)	0.678	0.669	0.658	0.324	0.215 (0.211–0.220)
DT	0.709 (0.691–0.730)	0.654	0.657	0.639	0.298	0.218 (0.211–0.227)
Random forest	0.823 (0.811–0.839)	0.755	0.74	0.734	0.470	0.180 (0.176–0.185)
CatBoost	**0.832 (0.819–0.845)**	**0.758**	**0.747**	**0.738**	**0.480**	0.169 (0.165–0.174)
LightGBM	0.820 (0.805–0.832)	0.752	0.736	0.730	0.462	**0.168 (0.162–0.174)**
XGBoost	0.810 (0.796–0.825)	0.755	0.736	0.732	0.465	**0.168 (0.162–0.174)**
**External validation**						
Logistic regression	0.651 (0.633–0.667)	0.593	0.645	0.578	0.257	0.330 (0.320–0.340)
SVM	0.710 (0.693–0.727)	0.567	0.639	0.558	0.249	0.251 (0.246–0.258)
Decision tree	0.643 (0.623–0.666)	0.580	0.616	0.561	0.206	0.261 (0.252–0.270)
Random forest	0.750 (0.735–0.767)	0.674	0.665	0.635	0.298	0.214 (0.210–0.218)
CatBoost	**0.757 (0.739–0.776)**	**0.691**	**0.692**	**0.655**	**0.345**	**0.207 (0.202–0.211)**
LightGBM	0.751 (0.736–0.766)	0.678	0.691	0.647	0.340	0.216 (0.209–0.225)
XGBoost	0.748 (0.733–0.763)	0.681	0.681	0.645	0.326	0.214 (0.207–0.222)

AUROC, The area under the receiver operating characteristic curve; CI, confidence interval; MCC, matthews correlation coefficient; SVM, support vector machine; CatBoost, categorical boosting; LightGBM, light gradient boosting machine; XGBoost, eXtreme gradient boosting. Bold values are to highlight how well the model performed on a certain metric.

**FIGURE 4:**
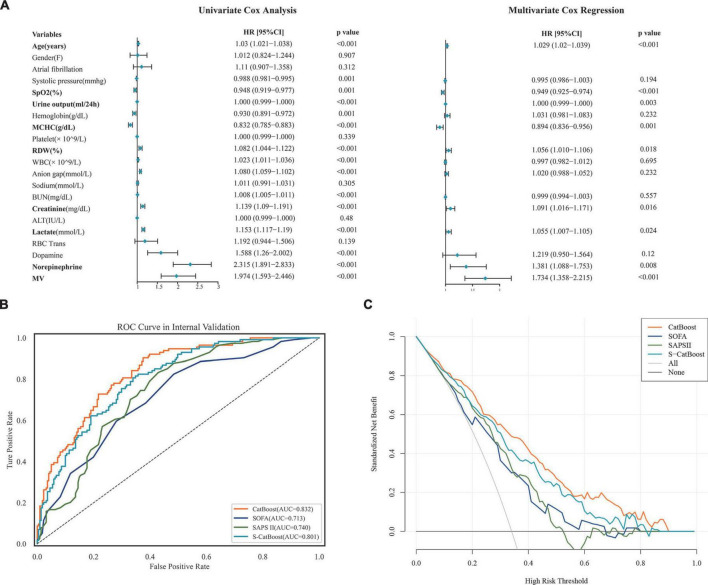
Model development and performance for S-CatBoost. **(A)** Univariate and multivariate Cox analyses of 21 features used to construct model; **(B)** ROC curve of CatBoost, S-CatBoost, SAPS II score, and SOFA score in internal validation; **(C)** DCA curve of CatBoost, S-CatBoost, SAPS II score and SOFA score in internal validation. Catboost, Categorical Boosting; S-Catboost, simplified Catboost model; AUC, area under the curve; DCA, Decision curve analysis; SAPS II, simplified acute physiology score II; SOFA, SOFA Score; SPO2, saturation of pulse oxygen; MCHC, mean corpuscular hemoglobin concentration; WBC, white blood cell count; BUN, blood urea nitrogen; RDW, red blood cell distribution width; RBC trans, red blood cell transfusion; ALT, alanine transaminase; MV, mechanical ventilation.

The entire 21 features were subjected to the COX univariate analysis, and those with differences (*P* < 0.05) were included in the multivariate COX analysis. Variables included in the multivariate analysis were checked for collinearity, and we found no significant collinearity (Supplementary Table 8). The results suggested that age, saturation of pulse oxygen (SpO2), mean corpuscular hemoglobin concentration (MCHC), lactate, urine output, red blood cell distribution width (RDW), creatinine, norepinephrine, and MV were independent factors for 30-day mortality in HH-HF patients (Figure 4A). Based on these independent factors, we developed a simplified Catboost model (S-Catboost), which achieved an AUC of up to 0.801 (95%CI, 0.786–0.812) in internal validation and outperformed SAPS II (AUC, 0.740; 95%CI, 0.723–0.756) and SOFA scores (AUC, 0.713; 95%CI, 0.696–0.729) (Figure 4B). In addition, in external validation, although the accuracy of the S-Catboost model decreased slightly compared with the full Catboost model, it still maintained a high level of discrimination with an AUC of 0.729 (95%CI, 0.711–0.745) (Supplementary Figure 7). Decision curve analysis (DCA) suggests that the Catboost and S-Catboost models can provide greater clinical utility than SAPS2 and SOFA scores (Figure 4C). The results of Delong’s test for each ROC curve are shown in Supplementary Table 9.

### Model interpretation

**FIGURE 5:**
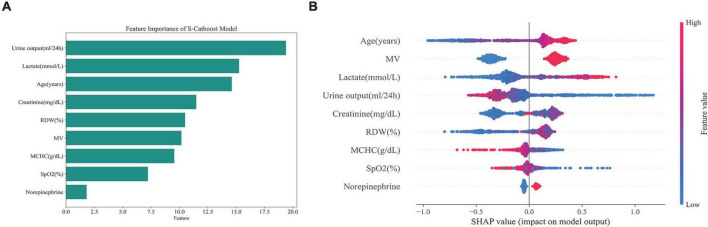
Feature importance ranking of S-Catboost model. **(A)** Feature importance ranking based on S-CatBoost; **(B)** feature importance ranking based on the SHAP value. The vertical axis is from top to bottom, and the importance of features decreases. The position of the point on the horizontal axis indicates the feature’s influence on the model’s predicted value, and the point’s color reflects the feature’s value. For the numerical variable, the blue and red points represent lower and higher values; for categorical variables, blue and red dots correspond to yes or no, respectively. S-Catboost, simplified Catboost model; MV, mechanical ventilation; SPO2, saturation of pulse oxygen; MCHC, mean corpuscular hemoglobin concentration; RDW, red blood cell distribution width.

The importance of features in the full Catboost model was largely consistent according to the model’s importance and the SHAP values, with MV, age, urine output, and lactate ranking highest (Supplementary Figure 8). According to feature importance in the S-Catboost model, the top five most important features were: urine output, lactate, age, creatinine, and RDW (Figure 5A). According to SHAP values, the top five features were age, MV, lactate, urine output, and creatinine (Figure 5B). The SHAP summary plot also illustrates features’ positive and negative effects on prediction results. The increase in age, MV, high lactic acid, and low urine output were associated with an increased risk of death, but the effects of creatinine and RDW on death risk do not follow a linear pattern (Figure 5B).

**FIGURE 6:**
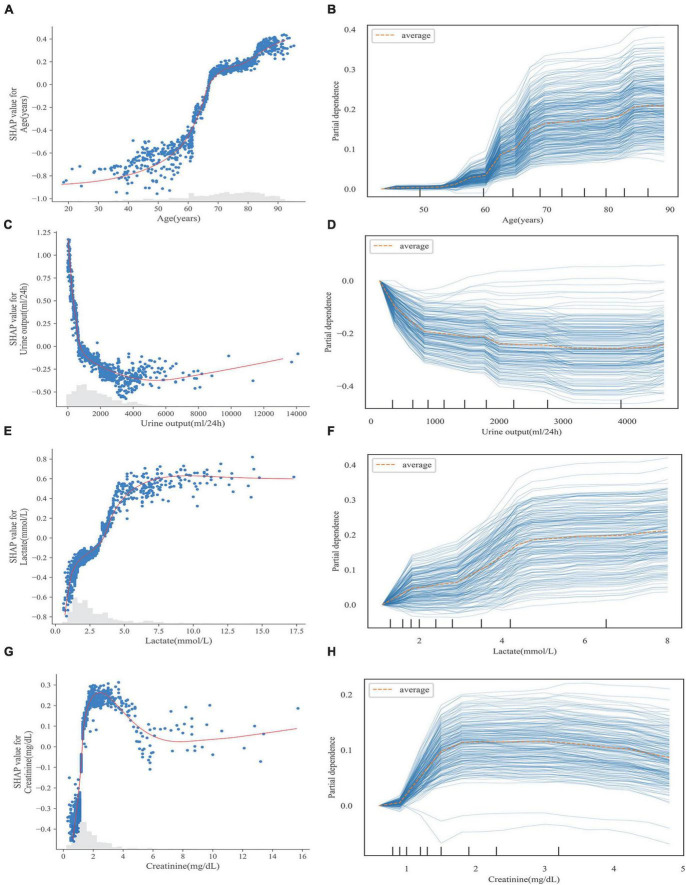
Scatter plot **(A,C,E,G)** and PDP **(B,D,F,G)** of continuous variables in S-CatBoost model. **(A,B)** Age; **(C,D)** urine output; **(E,F)** lactate; **(G,H)** creatinine. PDP, partial dependence plot; S-Catboost, simplified Catboost model.

Figure 6 illustrates the relationship between the four continuous variables of higher importance and the final predicted outcome in the S-CatBoost model. The role of individual features on the final prediction outcome can be directly observed in scatter plots based on the SHAP values. To further clarify the impact of particular features on the final predicted outcome, we also introduced PDP and individual conditional expectations (ICE) plots, another strategy for exploring the relationship between eigenvalues and predicted results. By visualizing the feature dependency for each case, the ICE plot shows the changing trend of the predicted outcome as the feature changes, while DPD calculates the mean level of the feature across all samples. The risk of death rapidly increased with age between the ages of 50 and 70, a trend that was more pronounced for ages between 60 and 70 (Figure 6A), while the trend began at 55 in the PDP, and the trend for ages 60–70 was consistent with the scatter plots (Figure 6B). It can be seen from Figure 6C that when urine output is less than 1,000 ml, the risk of death increases rapidly with decreasing urine output, and a similar trend can be seen in PDP (Figure 6D). The changing relationship between lactate values and the predicted outcome was consistent in Figures 6E,F. It appears that, below 5 mmol/L, the risk of death increased sharply with increasing lactate, and this trend slowed down between 2 and 3 mmol/L, but hardly increased at all after exceeding 7.5 mmol/L. Additionally, creatinine showed a relatively consistent trend in the PDP and scatter plots. When creatinine did not exceed 2 mg/dl, the risk increased with increasing creatinine. However, the risk began to decrease after creatinine exceeded 2 mg/dl, although this trend was more pronounced in the scatter plots (Figures 6G,H). The scatter and PDP plots for other continuous variables are shown in Supplementary Figure 9.

**FIGURE 7:**
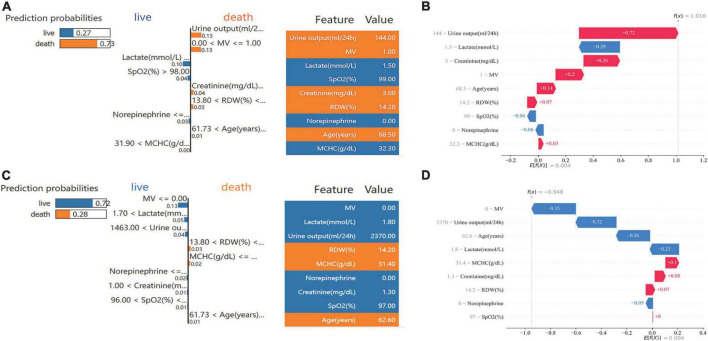
Specific prediction and interpretation of the S-CatBoost model for two patients. **(A,C)** Individual prediction interpretation based on SHAP; **(B,D)** individual prediction interpretation based on the LIME method. S-Catboost: simplified Catboost model. SHAP, Shapley Additive Explanations; LIME, Local Interpretable Model-agnostic Explanations; MV, mechanical ventilation; SPO2, saturation of pulse oxygen; MCHC, mean corpuscular hemoglobin concentration; RDW, red blood cell distribution width.

Examples of applying the S-Catboost model for risk prediction in individual patients are shown in Figure 7. The predicted outcome for the first patient was the occurrence of 30-day death. Figures 7A,B describe the interpretations of the predicted outcome based on the SHAP and LIME, respectively: according to both interpretations, oliguria, high creatinine, and MV were the most important factors in determining a patient’s death within 30 days. The second patient was predicted to survive within 30 days. The absence of the need for MV, higher urine output and lower lactate levels were the most important determinants of their survival, as seen in SHAP and LIME (Figures 7C,D).

## Discussion

In this study, the MIMIC database was retrospectively reviewed for HF patients and HF patients with HH. While it was believed previously that HH was a rare hepatic complication in patients with HF, the incidences of HH in HF patients admitted to the ICU were not low, with 6.5% occurring in the MIMIC dataset and 5.5% in the EICU dataset. This study found that this incidence is even higher than that reported in several previous large-scale studies of patients in ICU (11, 30). This study revealed 30-day mortality of 33.3% for HF patients with HH, whereas Van den Broecke et al. reported a 28-day mortality of 40.1% for HH patients in the HF subgroup, which was slightly higher than this study (11). The 1-year mortality rate of HF patients with combined HH was as high as 51%, which is in line with the 2-year mortality rate of 55% for HH reported by Taylor et al. (15). Furthermore, the effect of HH on survival impact in HF patients is clarified in this study: whether PSM was performed to eliminate baseline differences or not, HH increased both short—and long-term mortality in HF patients.

Despite the extremely high mortality rate associated with HH, there are currently no very effective therapies available. A case of rapid improvement of liver function in HH patients by artificial liver therapy was reported in the study by Drolz et al. (35). In another prospective study conducted by Drolz et al., statin use in prehospital hospitals was found to reduce 28-day mortality in HH patients (36). Nonetheless, these studies were performed on a small scale, and the effectiveness of these treatments on the survival of HH patients was not well documented, nor is there sufficient evidence to recommend their regular use. These treatments’ efficacy in improving HH patients’ survival requires more careful consideration. It is currently the main treatment strategy for HH to correct the underlying disease status and primary cause, yet the mortality rate remains high (37). Clinical decisions can be made more effective provided that clinicians can use predictive tools to identify high-risk patients early and optimize their clinical management. Horvatits et al. found that indocyanine green plasma display rate (ICG-PDR) could be used as a predictor of the prognosis of HH patients. However, ICG-PDR acquisition, dependent on an intravenous injection into the central line and good peripheral perfusion, is an invasive operation, and its practical application in the clinic is challenging. In addition, the sample size of this study was relatively small, comprising only 57 patients with HH (38). A wide variety of machine learning methods have been successfully applied to medicine with great flexibility and precision and have been employed in early diagnosis, risk stratification, and trend prediction. It has previously been reported that machine learning models have been used in cardiology for predicting survival in patients with HF and its complications (39–41). In this study, machine learning techniques were applied to develop the first prediction model that could be used to predict the mortality risk in HF patients with HH accurately. Despite its precise predictions, the “black box” nature of the prediction process hinders its generalization for practical use. Recently, the study of interpretability, which facilitates the transparency of the predictive process, has become an important focus in the field of machine learning, and progress has been made in many areas of this study (42). It should be noted, however, that interpretations of model results generated on different theoretical bases may vary considerably, and interpretations based on only one theoretical approach may be subject to some contingencies, resulting in unconvincing interpretations. Several interpretability techniques were used to interpret the model to reduce the chances of this occurring. A combination of both forms of interpretation was used at each level of the model (global, feature, and individual) to ensure stability and objectivity in the interpretation of the results. Moreover, the S-CatBoost model developed based on Cox multivariate analysis is relatively easy to understand and use, and it also performs well on EICU datasets consisting of multiple ICUs in terms of stability and generalizability.

The multivariate Cox analysis showed that age was an independent risk factor for 30-day mortality in HF patients with HH and an important factor in the prediction model. According to Aboelsoud et al., a retrospective study of 563 HH patients reached the same conclusion, hospital mortality increased by 19% for every 5-year increase in the age of HH patients (30); Furthermore, both Jonsdottir’s and Fuhrmann’s studies revealed that age was an independent risk factor for HH patient mortality (29, 43). Both models’ interpretability suggests that the risk of death increases with age, and this trend is highly noticeable in individuals aged 60–70. Several studies have demonstrated a close connection between acute kidney injury (AKI) and prognosis in HH patients, with approximately 67–81% of HH patients having comorbid AKI and AKI being an independent risk factor for mortality in HH patients (30, 44, 45). As it is very difficult to accurately assess whether a patient has developed AKI within 24 h of admission to the ICU, AKI was not directly included in this study. However, the impact of AKI can still be reflected in urine output and creatinine taken as independent risk factors for prognosis in HH patients, and we found that both decreased urine output and higher creatinine were strongly associated with increased mortality risk, while oliguria and high creatinine were among the most prominent markers of AKI. In AKI, imbalances in mechanisms such as glomerulo-tubular homeostasis, sodium excretion regulation, and others can cause hemodynamic disturbances throughout the body, leading to or aggravating the hypoxic state of the body, which will undoubtedly cause serious hypoxic damage to the liver in severe congestions. MV and high lactate levels are also manifestations of hypoxia’s impact on hemodynamics in HH patients. A higher proportion of patients in the non-survivor group required MV on the first day of admission, and the use of MV increased mortality risk, a finding that was also reported by Chavez Tapia et al. (46). Typically, lactate elevation results from improved anaerobic metabolic processes triggered by tissue hypoxia, which can be observed in many severe diseases and is strongly associated with prognosis. Lactate also plays a substantial role in the prognosis of HF patients with HH, with higher lactate causing a greater risk of death. According to Jonsdottir S’s study, elevated lactate was also a relevant prognostic factor in HH (29).

We explored the survival impact of HH in HF patients and applied a machine learning model to predict prognosis. This was the first tool to accurately predict the prognosis of HF patients with HH using clinical information that was quite convenient to obtain. Nevertheless, there are some limitations to our study. First, we used relatively low transaminase criteria for HH diagnosis, which, while increasing the sensitivity of the study population, also undermines specificity. Due to the limited follow-up timeframe in the MIMIC dataset, we were only able to analyze survival within 1 year, while the longer-term survival impact of HH on HF patients is unclear. Additionally, we only examined the overall survival impact, and a detailed analysis of survival impacts across different periods is needed. This study was limited to HF patients with HH within the ICU due to the lack of direct information about cardiac functional grades. The morbidity and prognosis of HF patients in different cardiac functional grades were not examined. As a result of the higher rate of missing data, cardiac-related indicators such as cardiac output, BNP, and myocardial markers were not included in the study, resulting in the loss of some potentially useful data. All of the predictive features used in this study were acquired within 24 h of admission to the ICU, thus allowing for early prediction. However, it must be acknowledged that these variables have a limited impact on subsequent morbidities and longer-term outcomes.

## Conclusion

In patients with HF, HH is an independent risk factor for increased short- and long-term mortality. The machine learning model effectively predicted 30-day mortality in HF with HH with good generalization ability. Multiple interpretability techniques can increase the transparency of the model and the stability of the interpretation, which will facilitate the understanding of the model and its application in practice.

## Data availability statement

Publicly available datasets were analyzed in this study. This data can be found here: https://physionet.org/. The code in this study is available on GitHub (https://github.com/sunrun519/MLmodel_for_CFHH).

## Ethics statement

The studies involving human participants were reviewed and approved by the Institutional Review Board at the Beth Israel Deaconess Medical Center. Written informed consent for participation was not required for this study in accordance with the national legislation and the institutional requirements.

## Author contributions

ZH and LQ: conception and design. HJ: administrative support. RS and XW: provision of study materials or patients. HJ, XW, YD, and WY: collection and assembly of data. RS, XW, and YY: data analysis and interpretation. All authors contributed to the article and approved the submitted version.

## Abbreviations

HH: hypoxic hepatitis
HF: heart failure
MIMIC: Medical Information Mart for Intensive Care
ICU: intensive care unit
PSM: Propensity Score Matching
RFE: feature recurrence elimination
HPO: hyper-parameter optimization
SHAP: Shapley Additive Explanations
LIME: Local Interpretable Model-agnostic Explanations
PDP: Partial Dependence Plot
Catboost: Categorical Boosting
COVID-19: coronavirus disease 2019
EICU CRD/EICU: The EICU Collaborative Database
ALT: alanine transaminase
AST: aspartate transaminase
SQL: structured language
MV: mechanical ventilation
RRT: renal replacement therapy
RBC: red blood cell
FFP: fresh frozen plasma
SAPS II: simplified acute physiology score II
SOFA: sequential organ failure
RFE-CV: feature recursive elimination- cross-validation
XGBoost: eXtreme Gradient Boosting
SMOTE: Synthetic Minority Oversampling Technique
LR: logistics regression
DT: decision tree
SVM: support vector machine
RF: random forest
CatBoost: categorical boosting
LightGBM: light gradient boosting machine
ROC: receiver operating characteristic curve
AUC: area under the curve
MCC: Matthews correlation coefficient
BUN: blood urea nitrogen
SCR: serum creatinine
SPO2: saturation of pulse oxygen
MCHC: mean corpuscular hemoglobin concentration
RDW: red blood cell distribution width
S-Catboost: simplified Catboost model
DCA: Decision curve analysis
ICE plot: individual conditional expectations plot
ICG-PDR: indocyanine green plasma display rate
AKI: acute kidney injury.
