# Supplementary material for: Prediction of 30-day mortality in heart failure patients with hypoxic hepatitis: Development and external validation of an interpretable machine learning model
Source: Front Cardiovasc Med. 2022 Oct 28;9:1035675. doi: 10.3389/fcvm.2022.1035675 (PMC9649827; doi:10.3389/fcvm.2022.1035675)
Supplement: Supplementary file 1 [file Data_Sheet_1.PDF]

Supplementary information to:

Title Prediction of 30-Day Mortality in Heart Failure Patients with Hypoxic Hepatitis: Development and External Validation of an Interpretable Machine Learning Model

Authors

Run Sun [1,2]

Xue Wang[1,2]

Haiyan Jiang [1,3]

Yan Yan[1], Yansong Dong[2]

Wenxiao Yan[1], Xinye Luo[1,2]

Hua Miu[1], Lei Qi [1,2*]

Zhongwei Huang[1,2*]

Affiliations

[1]Medical School of Nantong University, Nantong University, Nantong, China;

[2] Department of Emergency Medicine, Affiliated Hospital of Nantong University, Nantong, China;

[3]Health Management Center, Affiliated Hospital of Nantong University, Nantong, China.

Corresponding Authors Information

Lei Qi：qilei723@ntu.edu.cn;

Zhongwei Huang：hzw889@163.com

Supplementary Materials

Table

Table S1. Disease excluded due to potential elevation of transaminase.

Table S2-1. Distribution of continuous variables of heart failure patients before and after filling in the MIMIC database.

Table S2-2. Distribution of continuous variables of heart failure patients with hypoxic hepatitis before and after filling in the MIMIC database.

Table S2-3. Distribution of continuous variables of heart failure patients with hypoxic hepatitis before and after filling the EICU database.

Table S3. Characteristics between heart failure patients with and without hypoxic hepatitis before and after PSM

Table S4. Characteristics between survivor and non-survivor groups of heart failure patients with hypoxic hepatitis

Table S5. Feature ranking during recursive feature elimination

Table S6. Hyperparameter search domains and final settings of seven model

Table S7. Performance of the models in a 10-fold cross-validation

Table S8. Collinearity checks for continuous variables were performed for multivariate Cox analysis

Table S9. Delong test of ROC curve

Figures

Figure S1. The screening process of patients and the main flow of the study.

Figure S2. Standardized mean difference between the heart failure patients with and without hypoxic hepatitis before and after PSM

Figure S3-1. Multivariate Cox analysis of 30-day mortality in patients with heart failure

Figure S3-2. Multivariate Cox analysis of 90-day mortality in patients with heart failure

Figure S3-3. Multivariate Cox analysis of 180-day mortality in patients with heart failure

Figure S3-4. Multivariate Cox analysis of 365-day mortality in patients with heart failure

Figure 4. Number of features and corresponding accuracy scores in feature recursive elimination cross-validation

Figure5-1. Hyperparameter optimization process for the logistic regression model

Figure5-2. Hyperparameter optimization process for the decision tree model

Figure5-3. Hyperparameter optimization process for the support vector machine model

Figure5-4. Hyperparameter optimization process for the Random Forest model

Figure5-5. Hyperparameter optimization process for the eXtreme Gradient Boosting model

Figure5-6. Hyperparameter optimization process for the light gradient boosting machine model

Figure S6. Learning curve of each model in the training process with final parameters

Figure S7. ROC curve of S-CatBoost in external validation.

Figure S8. Feature importance ranking of the Catboost model.

Figure S9. Scatter plot and PDP of other continuous variables in S-CatBoost model.

Table S1. Disease excluded due to potential elevation of transaminase.

	MIMIC-IV database		MIMIC-III database	EICU database
	**ICD9**	**ICD10**	**ICD9**	**IDC9/10 code**
Viral hepatitis	701;7020;7022;7030;7031;7032;7044;7054;7070;7071;5731;	B159；B179；B181; B182; B188;B1910;B1920;B251;O9843;	7054;7070;70307044;7032;7071;701;7051;7041;7022;5731;	573.1;070.51; B17.1;070.30;573.1; B19.10;573.1; B17.9;573.1;070.30; B17.1
Hepatic failure	570	K7040K7041; K7111;K7200K7201;K7210; K7290;K7291;K9182;	570	570; K72.9;573.9; K76.9;573.9;K76.9; 286.7;D68.4;038.9;570;R65.20;K72.0;573.9;K76.9;570;K72.0;790.6; R73.9;570; K72.0
Chronic liver disease and Cirrhosis	5712;5715;5716	K7030; K7031;K717;K743;K745;K7460;K7469;	57155712;5728;5710;5716;	573.9; K76.9;571.5;K74.60;572.2;789.5;R18.8; 571.2;K70.3;573.9; K76.9;276.1;E87.1;572.4;K76.7;573.9;K76.9;572.4;K76.7;571.5;K74.69
Toxic liver disease and Autoimmune hepatitis	57142	K712;K713;K716;K718;K719;K754	57142	573.3; T71.2;571.1; K70.1
Hepatic surgery; Hepatic injury and Hepatic infarction	5734;86400;86401; 86405;99682;V427	K763;K765;S36112A;S36113A;T8640;T8641;T8642;T8643;Z944	86401;5734;86403; 99682; V427	864.00; S36.11;864.05
Cholangitis	5761	K8030K8031;K8032;K8033; K830;K8309	5761	576.1; K83.0
Pancreatitis	5770;5771	K8500;K851;K8510;K8512; K852;K8520K8521;K853;K8580;K8581;K859;K8590;K8591;K860;K861;	5770;5771	577.0; K85; K85.9;577.1; K86.1;577.8; K85.9;518.81; J80
Rhabdomyolysis	72888	M6282	72888	359.89; G72.89;584.9; N17.9;728.89;276.7; E87.5

Diagnoses in the mimic database are based on a structured language with ICD9 and ICD10 disease codes in the MIMIC-IV database and only ICD-9 in MIMIC-III. The data in the EICU are based on an unstructured language, consisting of ICD9 and ICD10 simultaneously forming a diagnostic ring. MIMIC: Medical Information Mart for Intensive Care; EICU: eICU Collaborative Research Database; ICD: International Classification of Diseases.

Table S2-1: Distribution of continuous variables of heart failure patients before and after filling in the MIMIC database.

Variable	Miss Rate (%)	Procedure	Before Filling				After Filling			
			Mean+SD	Q25	Q75	CV (%)	Mean+SD	Q25	Q75	CV (%)
HR, bpm	0.35	Filling	84.43±15.44	74	94	18.3	84.43±15.43	74	94	18.3
SBP, mmHg	0.58	Filling	117±16	105	126	14.0	1175±16	105	126	14.0
DBP, mmHg	0.58	Filling	59.17±10.55	52	65	17.8	59.18±10.54	52	65	17.8
RR, bpm	0.39	Filling	19.72±3.86	17	22	19.6	19.72±3.86	17	22	19.6
Temperature, ℃	4.92	Filling	36.77±0.55	36.5	37.1	1.5	36.77±0.54	36.5	37.1	1.5
SPO_2,%	0.38	Filling	96.82±2.10	96	98	2.2	96.84±2.13	96	98	2.2
UO, ml/24h	2.93	Filling	1896±1349	1000	2545	71.1	1879±1341	993	2520	71.4
HCT, %	1.14	Filling	31.65±6.25	27.2	35.7	19.7	31.65±6.24	27.2	35.7	19.7
HB, g/dL	1.56	Filling	10.43±2.09	8.9	11.8	20.0	10.43±2.08	8.9	11.7	20.0
MCHC, g/dL	1.59	Filling	32.96±1.71	31.9	34.1	5.2	32.94±1.71	31.9	34.1	5.2
MCV,fL	1.60	Filling	90.58±6.86	86	95	7.6	90.56±6.85	86	95	7.6
Platelet, × 10^9/L	1.35	Filling	216±106	146	263	49.2	216±106	147	263	48.9
RBC, × 10^12/L	1.59	Filling	3.52±0.73	3.0	4.0	20.7	3.52±0.73	3.0	4.0	20.6
RDW, %	1.64	Filling	15.34±2.10	13.9	16.3	13.7	15.35±2.09	13.9	16.3	13.6
WBC,× 10^9/L	1.43	Filling	12.43±10.31	7.9	14.8	82.9	12.43±10.25	7.9	14.8	82.4
**ALT, IU/L**	62.63	Eliminate								
**AST, IU/L**	62.55	Eliminate								
**TBIL, g/dL**	62.32	Eliminate								
Anion gap, mmol/L	1.98	Filling	14.55±3.89	12	17	26.7	14.52±3.88	12	17	26.7
Bicarbonate, mmol/L	0.92	Filling	24.24±5.00	21	27	20.6	24.24±4.98	21	27	20.6
Chloride, mmol/L	0.90	Filling	103.76±6.67	100	108	6.4	103.75±6.65	100	108	6.4
Calcium, mmol/L	12.93	Filling	8.41±0.82	8	9	9.8	8.38±0.79	8	9	9.5
Glucose, g/dL	2.18	Filling	149±76	106	167	50.8	149±75	106	166	50.5
Sodium, mmol/L	1.12	Filling	138.53±4.97	136	141	3.6	138.54±4.95	136	141	3.6
Potassium, mmol/L	1.53	Filling	4.27±0.72	3.8	4.6	16.9	4.27±0.72	3.8	4.6	16.9
BUN,mg/dL	0.75	Filling	32.93±23.60	17	41	71.7	32.95±23.56	17	41	71.5
SCr,mg/dL	0.69	Filling	1.62±1.54	0.8	1.8	95.0	1.63±1.54	0.8	1.8	94.8
PT,s	13.68	Filling	17.27±9.48	13.2	17.4	54.9	17.32±9.16	13.2	17.6	52.9
INR	13.68	Filling	1.64±1.10	1.2	1.7	67.1	1.65±1.06	1.2	1.7	64.4
**PO_2,mmHg**	34.65	Eliminate								
**PCO_2, mmHg**	34.66	Eliminate								
**PH**	33.76	Eliminate								
**Lactate,mmol/L**	46.56	Eliminate								
SAPSII	0.42	Filling	39.58±12.67	31	47	32.0	39.58±12.66	31	47	32.0
SOFA	0.29	Filling	5.82±3.10	4	8	53.2	5.83±3.09	4	8	53.1

MIMIC medical information mart for intensive care; CV: Coefficient of Variation; HR: heart rate; SBP: systolic pressure; DBP: diastolic pressure; RR: respiratory rate; SPO_2:saturation of pulse oxygen; UO: urine output; HCT: hematocrit; HB: hemoglobin; MCHC: mean corpuscular hemoglobin concentration; MCV: Mean Corpuscular Volume; RBC: red blood cell count; RDW: red blood cell distribution width; WBC: white blood cell; ALT: alanine transaminase; AST: aspartate transaminase; TBIL: total bilirubin; BUN: blood urea nitrogen; SCr: serum creatinine; PT: prothrombin time; INR: international normalized ratio; PaO_2: arterial partial pressure of oxygen; $PaCO_2$: arterial partial pressure of carbon dioxide; SOFA: sequential organ failure assessment; SAPS: simplified acute physiology score II.

Table S2-2: Distribution of continuous variables of heart failure patients with hypoxic hepatitis before and after filling in the MIMIC database.

Variable	Miss Rate (%)	Procedure	Before Filling				After Filling			
			Mean+SD	Q25	Q75	CV (%)	Mean+SD	Q25	Q75	CV (%)
HR, bpm	0.35	Filling	84.43±15.44	74	94	18.3	84.43±15.43	74	94	18.3
SBP, mmHg	0.58	Filling	117±16	105	126	14.0	1175±16	105	126	14.0
DBP, mmHg	0.58	Filling	59.17±10.55	52	65	17.8	59.18±10.54	52	65	17.8
RR, bpm	0.39	Filling	19.72±3.86	17	22	19.6	19.72±3.86	17	22	19.6
Temperature, ℃	4.92	Filling	36.77±0.55	36.5	37.1	1.5	36.77±0.54	36.5	37.1	1.5
SPO_2, %	0.38	Filling	96.82±2.10	96	98	2.2	96.84±2.13	96	98	2.2
UO, ml/24h	2.93	Filling	1896±1349	1000	2545	71.1	1879±1341	993	2520	71.4
HCT, %	1.14	Filling	31.65±6.25	27.2	35.7	19.7	31.65±6.24	27.2	35.7	19.7
HB, g/dL	1.56	Filling	10.43±2.09	8.9	11.8	20.0	10.43±2.08	8.9	11.7	20.0
MCHC, g/dL	1.59	Filling	32.96±1.71	31.9	34.1	5.2	32.94±1.71	31.9	34.1	5.2
MCV, fL	1.60	Filling	90.58±6.86	86	95	7.6	90.56±6.85	86	95	7.6
Platelet, × 10^9/L	1.35	Filling	216±106	146	263	49.2	216±106	147	263	48.9
RBC, × 10^12/L	1.59	Filling	3.52±0.73	3.0	4.0	20.7	3.52±0.73	3.0	4.0	20.6
RDW, %	1.64	Filling	15.34±2.10	13.9	16.3	13.7	15.35±2.09	13.9	16.3	13.6
WBC, × 10^9/L	1.43	Filling	12.43±10.31	7.9	14.8	82.9	12.43±10.25	7.9	14.8	82.4
**ALT, IU/L**	62.63	Eliminate								
**AST, IU/L**	62.55	Eliminate								
**TBIL, g/dL**	62.32	Eliminate								
Anion gap, mmol/L	1.98	Filling	14.55±3.89	12	17	26.7	14.52±3.88	12	17	26.7
Bicarbonate, mmol/L	0.92	Filling	24.24±5.00	21	27	20.6	24.24±4.98	21	27	20.6
Chloride, mmol/L	0.90	Filling	103.76±6.67	100	108	6.4	103.75±6.65	100	108	6.4
Calcium, mmol/L	12.93	Filling	8.41±0.82	8	9	9.8	8.38±0.79	8	9	9.5
Glucose, g/dL	2.18	Filling	149±76	106	167	50.8	149±75	106	166	50.5
Sodium, mmol/L	1.12	Filling	138.53±4.97	136	141	3.6	138.54±4.95	136	141	3.6
Potassium, mmol/L	1.53	Filling	4.27±0.72	3.8	4.6	16.9	4.27±0.72	3.8	4.6	16.9
BUN, mg/dL	0.75	Filling	32.93±23.60	17	41	71.7	32.95±23.56	17	41	71.5
SCr, mg/dL	0.69	Filling	1.62±1.54	0.8	1.8	95.0	1.63±1.54	0.8	1.8	94.8
PT, s	13.68	Filling	17.27±9.48	13.2	17.4	54.9	17.32±9.16	13.2	17.6	52.9
INR	13.68	Filling	1.64±1.10	1.2	1.7	67.1	1.65±1.06	1.2	1.7	64.4
**PO_2,mmHg**	34.65	Eliminate								
**PCO_2, mmHg**	34.66	Eliminate								
**PH**	33.76	Eliminate								
**Lactate,mmol/L**	46.56	Eliminate								
SAPSII	0.42	Filling	39.58±12.67	31	47	32.0	39.58±12.66	31	47	32.0
SOFA	0.29	Filling	5.82±3.10	4	8	53.2	5.83±3.09	4	8	53.1

MIMIC Medical Information Mart for Intensive Care; CV: Coefficient of Variation; HR: heart rate; SBP: systolic pressure; DBP: diastolic pressure; RR: respiratory rate; SPO_2:saturation of pulse oxygen; UO: urine output; HCT: hematocrit; HB: hemoglobin; MCHC: mean corpuscular hemoglobin concentration; MCV: Mean Corpuscular Volume; RBC: red blood cell count; RDW: red blood cell distribution width; WBC: white blood cell; ALT: alanine transaminase; AST: aspartate transaminase; TBIL: total bilirubin; BUN: blood urea nitrogen; SCr: serum creatinine; PT: prothrombin time; INR: international normalized ratio; PaO_2: arterial partial pressure of oxygen; $PaCO_2$: arterial partial pressure of carbon dioxide; SOFA: sequential organ failure assessment; SAPS II: simplified acute physiology score II.

Table S2-3: Distribution of continuous variables of heart failure patients with hypoxic hepatitis before and after filling the EICU database.

Variable	Miss Rate (%)	Procedure	Before Filling				After Filling			
			Mean+SD	Q25	Q75	CV(%)	Mean+SD	Q25	Q75	CV (%)
Age, year	0.00	N/A								
SBP, mmHg	0.00	N/A								
SPO_2, %	0.00	N/A								
UO, ml/24h	28.34	Filling	1634±1566.9	537	2373	95.89	1584±1551.8	509	2315	97.99
HB, g/dL	3.58	Filling	11.4±2.49	9.4	13.2	21.85	11.4±2.5	9.4	13.2	21.91
MCHC, g/dL	4.89	Filling	32.5±1.52	31.8	33.6	4.68	32.5±1.5	31.8	33.6	4.62
Platelet, × 10^9/L	3.91	Filling	197±87.72	138	246	44.52	198±88.67	140	246	44.77
RDW, %	9.12	Filling	16.3±2.94	14.4	17.4	18.01	16.5±3.03	14.5	17.6	18.43
WBC, × 10^9/L	3.91	Filling	13.6±6.68	9.3	16.9	49.10	13.5±6.59	9.3	16.7	48.76
Anion gap, mmol/L	19.22	Filling	14±5.29	10	16	38.21	14±5.35	10	16	38.78
Sodium, mmol/L	1.63	Filling	136±5.84	133	140	4.28	136±5.86	133	140	4.30
BUN, mg/dL	1.95	Filling	41±25.31	23	54	61.97	41±25.23	23	54	61.66
SCr, mg/dL	2.28	Filling	2.1±1.49	1.1	2.6	70.09	2.1±1.5	1.1	2.6	69.88
ALT, IU/L	12.70	Filling	401±706.5	42	408	176.09	407±728.6	41	397	179.16
Lactate, mmol/L	29.97	Filling	4.1±3.46	1.7	5.7	84.49	3.7±3.20	1.6	4.8	85.36

EICU: EICU Collaborative Database; CV: Coefficient of Variation; SBP: systolic pressure; SPO_2:saturation of pulse oxygen; UO: urine output; HB: hemoglobin; MCHC: mean corpuscular hemoglobin concentration; RDW: red blood cell distribution width; WBC: white blood cell; BUN: blood urea nitrogen; SCr: serum creatinine; ALT: alanine transaminase.

Table S3: Characteristics between heart failure patients with and without hypoxic hepatitis before and after PSM

Variables	Before matching			After matching		
	NO-Hypoxic hepatitis (n = 16100)	Hypoxic hepatitis (n=1114)	p	NO-Hypoxic hepatitis (n=1096)	Hypoxic hepatitis (n=1096)	p
**All-cause mortality**						
30-day	2509(15.58)	378(33.93)	<0.001	228(20.80)	367(33.49)	<0.001
90-day	3660(22.73)	456(40.93)	<0.001	301(27.46)	445(40.60)	<0.001
180-day	4437(27.56)	522(46.86)	<0.001	345(31.48)	511(46.62)	<0.001
365-day	5506(34.20)	565(50.72)	<0.001	411(37.50)	554(50.55)	<0.001
**Demography**						
Age	76.1(65.9,84.1)	72.5(61.7,81.0)	<0.001	71.8(60.9,81.3)	72.6(61.8,81.2)	0.471
Gender(F)	7590(47.15)	444(39.86)	<0.001	438(39.96)	437(39.87)	0.965
**Comorbidity**						
Hypertension, %	11483(71.33)	713(64.00)	<0.001	686(62.59)	702(64.05)	0.478
Dyslipidemia, %	7034(43.69)	481(43.18)	0.738	480(43.80)	471(42.97)	0.698
Diabetes, %	6252(38.83)	429(38.51)	0.83	408(37.23)	422(38.50)	0.538
Coronary surgery history, %	3369(20.93)	238(21.36)	0.728	223(20.35)	235(21.44)	0.528
Old myocardial infarction, %	2049(12.73)	153(13.73)	0.331	148(13.50)	151(13.78)	0.852
Cardiomyopathy, %	2024(12.57)	208(18.67)	<0.001	234(21.35)	202(18.43)	0.087
Atrial fibrillation, %	7853(48.78)	536(48.11)	0.668	524(47.81)	527(48.08)	0.898
Chronic pulmonary disease, %	5853(36.36)	328(29.44)	<0.001	321(29.29)	324(29.56)	0.888
Chronic kidney disease, %	5137(31.91)	312(28.01)	0.007	313(28.56)	306(27.92)	0.74
Peripheral vascular diseases, %	2495(15.50)	187(16.79)	0.251	177(16.15)	184(16.79)	0.687
Cerebrovascular diseases, %	2111(13.11)	121(10.86)	0.031	126(11.50)	119(10.86)	0.635
Hypothyroidism, %	2506(15.57)	156(14.00)	0.163	148(13.50)	154(14.05)	0.71
Cancer, %	1717(10.67)	127(11.40)	0.443	127(11.59)	127(11.59)	1.000
**Vital signs**						
Heart Rate, bpm	83(73,94)	87(76,99)	<0.001	87(76,99)	87(76,99)	0.617
Systolic Pressure, mmHg	115(106,126)	108(100,118)	<0.001	108(101,118)	108(100,118)	0.692
Diastolic Pressure, mmHg	58(52,65)	60(53,67)	<0.001	60(54,67)	60(53,67)	0.45
Respiratory Rate, bpm	19(17,22)	20(18,23)	<0.001	20(17,23)	20(18,23)	0.78
Temperature, ℃	36.75(36.5,37.1)	36.76(36.4,37.1)	0.462	36.78(36.5,37.1)	36.77(36.4,37.1)	0.307
SpO_2, %	97(96,98)	97(96,98)	0.902	97(96,98)	97(96,98)	0.195
Urine output, ml/24h	1630(1005,2525)	1463(767.3,2465)	<0.001	1597(851,2604)	1470(776,2463)	0.151
**Laboratory tests**						
Hematocrit, %	31.0(27.1,35.5)	33.9(28.8,38.8)	<0.001	33.2(28.5,38.1)	33.7(28.7,38.7)	0.436
Hemoglobin, g/dL	10.2(8.9,11.7)	11.1(9.4,12.8)	<0.001	11.0(9.4,12.6)	11.1(9.4,12.7)	0.446
MCHC, g/dL	33.0(31.9,34.1)	33.0(32.0,34.1)	0.736	33.0(31.8,34.1)	33.0(32.0,34.1)	0.607
MCV, fL	90.0(86.0,95.0)	91.0(86.0,95.0)	0.492	91.0(86.0,95.0)	91.0(86.0,95.0)	0.781
Platelet, × 10^9/L	198.0(146.0,262.0)	206.0(148.0,276.0)	0.024*	202.0(144.0,281.0)	206.0(148.0,278.8)	0.401
RBC, × 10^12/L	3.44(3.0,4.0)	3.72(3.2,4.3)	<0.001	3.69(3.2,4.2)	3.71(3.2,4.3)	0.509
RDW, %	14.9(13.9,16.3)	14.8(13.8,16.5)	0.147	14.9(13.8,16.5)	14.8(13.7,16.5)	0.402
WBC, × 10^9/L	10.7(7.9,14.6)	13.3(9.9,17.6)	<0.001	12.2(8.9,17.6)	13.2(9.8,17.5)	0.006
Anion gap, mmol/L	14.0(12.0,16.0)	16.0(14.0,19.0)	<0.001	16.0(14.0,19.0)	16.0(14.0,19.0)	0.927
Bicarbonate, mmol/L	24.0(21.0,27.0)	22.0(19.0,25.0)	<0.001	22.0(19.0,25.0)	22.0(19.0,25.0)	0.846
Chlorine, mmol/L	104.0(1.0,108.0)	103.0(99.0,107.0)	<0.001	103.0(99.0,107.0)	103.0(99.0,107.0)	0.764
Calcium, mmol/L	8.4(7.9,8.8)	8.3(7.8,8.8)	<0.001	8.4(7.8,8.8)	8.3(7.8,8.8)	0.064
Glucose, mg/dL	128.0(106.0,164.0)	147.5(118.0,198.0)	<0.001	140.0(111.0,197.0)	147.0(118.0,197.8)	0.018
Sodium, mmol/L	139.0(136.0,141.0)	138.0(135.0,140.0)	<0.001	138.0(135.0,141.0)	138.0(135.0,140.0)	0.877
Potassium, mmol/L	4.2(3.8,4.6)	4.3(3.9,4.8)	<0.001	4.3(3.9,4.8)	4.3(3.9,4.8)	0.323
BUN, mg/dL	25.0(17.0,40.0)	30.0(19.0,48.0)	<0.001	28.0(18.0,46.0)	29.0(19.0,48.0)	0.139
SCr, mg/dL	1.1(0.8,1.8)	1.3(1.0,2.1)	<0.001	1.3(0.9,2.1)	1.3(1.0,2.1)	0.487
PT, s	14.7(13.2,17.5)	15.5(13.7,19.8)	<0.001	15.3(13.5,18.9)	15.4(13.6,19.7)	0.199
INR	1.3(1.2,1.7)	1.5(1.2,1.9)	<0.001	1.4(1.2,1.9)	1.5(1.2,1.9)	0.173
**Score**						
SAPSII	38(31,46)	44(35,55)	<0.001	43.(34,53)	44.(35,55)	0.225
SOFA	5(4,8)	7(5,10)	<0.001	7. (5,10)	7. (5,10)	0.615
**Treatment**						
FFP Trans, %	1207(7.50)	138(12.39)	<0.001	136(12.41)	130(11.86)	0.695
RBC Trans, %	3548(22.04)	245(21.99)	0.972	220(20.07)	238(21.72)	0.344
Dopamine, %	913(5.67)	219(19.66)	<0.001	195(17.79)	213(19.43)	0.323
Epinephrine, %	1229(7.63)	137(12.30)	<0.001	121(11.04)	126(11.50)	0.736
Norepinephrine, %	2564(15.93)	411(36.89)	<0.001	402(36.68)	398(36.31)	0.859
Phenylephrine, %	3089(19.19)	220(19.75)	0.646	195(17.79)	213(19.43)	0.323
RRT, %	725(4.50)	75(6.73)	0.001	66(6.02)	69(6.30)	0.790
MV, %	6627(41.16)	604(54.22)	<0.001	573(52.28)	588(53.65)	0.521

SpO2: saturation of pulse oxygen; MCHC: Mean corpuscular hemoglobin concentration; MCV: Mean Corpuscular Volume; RBC: Red blood cell count; RDW: Red blood cell distribution width; WBC: white blood cell count; BUN: blood urea nitrogen; SCr: Serum creatinine; PT: Prothrombin time; INR: international normalized ratio; SAPS II: The simplified acute physiology score II; SOFA: Sequential organ failure assessment; FFP Trans: fresh frozen plasma Transfusion; RBC trans: Red Blood Cell Transfusion; RRT: Renal replacement therapy; MV: mechanical ventilation.

Table S4: Characteristics between survivor and non-survivor groups of heart failure patients with hypoxic hepatitis

Variables	Survivor (n = 736)	Non-survivor (n = 378)	*P* value
**Demography**			
Age	70.850(59.9,79.2)	76.700(68.3,83.6)	<0.001
Gender(F)	293(39.81)	151(39.95)	0.965
**Comorbidity**			
Hypertension, %	471(63.99)	242(64.02)	0.993
Dyslipidemia, %	317(43.07)	164(43.39)	0.92
Diabetes, %	268(36.41)	161(42.59)	0.045
Coronary surgery history, %	140(19.02)	98(25.93)	0.008
Old myocardial infarction, %	94(12.77)	59(15.61)	0.193
Cardiomyopathy, %	143(19.43)	65(17.20)	0.365
Atrial fibrillation, %	346(47.01)	190(50.26)	0.303
Chronic pulmonary disease, %	209(28.40)	119(31.48)	0.285
Chronic kidney disease, %	186(25.27)	126(33.33)	0.005
Peripheral vascular diseases, %	103(13.99)	84(22.22)	0.001
Cerebrovascular diseases, %	75(10.19)	46(12.17)	0.315
Hypothyroidism, %	90(12.23)	66(17.46)	0.017
Cancer, %	72(9.78)	55(14.55)	0.018
**Vital signs**			
Heart Rate, bpm	87(76,98)	88(76,101)	0.311
Systolic Pressure, mmHg	109(101,119)	106(98,115)	<0.001
Diastolic Pressure, mmHg	61(54,69)	58(51,64)	<0.001
Respiratory Rate, bpm	20(18,23)	20(18,24)	0.045
Temperature, ℃	36.8(36.5,37.1)	36.7(36.3,37.1)	0.001
SpO_2, %	97(96,98)	97(95,99)	0.908
Urine output, ml/24h	1690(945,2694)	1019(435,1870)	<0.001
**Laboratory tests**			
Hematocrit, %	34.2(28.9,39.4)	33.0(28.6,37.4)	0.018
Hemoglobin, g/dL	11.3(9.5,13.0)	10.7(9.3,12.2)	<0.001
MCHC, g/dL	33.2(32.2,34.2)	32.6(31.4,33.9)	<0.001
MCV, fL	90(86,95)	91(87,96)	0.005
Platelet, × 10^9/L	208(156,275)	199(140,281)	0.068
RBC, × 10^12/L	3.8(3.2,4.4)	3.6(3.1,4.2)	0.002
RDW, %	14.5(13.5,16.2)	15.3(14.2,16.9)	<0.001
WBC, × 10^9/L	12.8(9.9,16.6)	14.4(9.9,19.3)	0.005
ALT, IU/L	140(70,273)	128(49,265)	0.017
AST, IU/L	262(164,426)	242(117,430)	0.024
TBIL, g/dL	0.8(0.5,1.3)	0.9(0.5,1.7)	0.01
Anion gap, mmol/L	16(14,18)	18(15,21)	<0.001
Bicarbonate, mmol/L	22(19,25)	21.000(18,24)	<0.001
Chlorine, mmol/L	103(99,107)	103(98,107)	0.698
Calcium, mmol/L	8.3(7.8,8.8)	8.3(7.7,8.7)	0.309
Glucose, mg/dL	143.5(115.0,191.0)	155.0(121.0,219.3)	0.003
Sodium, mmol/L	138(135,140)	138(135,141)	0.289
Potassium, mmol/L	4.3(3.9,4.7)	4.4(3.9,4.9)	0.036
BUN, mg/dL	26(18,44)	37(24,55)	<0.001

SCr, mg/dL	1.2(0.9,1.8)	1.6(1.1,2.6)	<0.001
PT, s	15.1(13.5,18.8)	16.1(14.0,20.6)	<0.001
INR	1.4(1.2,1.8)	1.5(1.3,2.0)	<0.001
PO_2,mmHg	102(68,179)	98(62,200)	0.42
PCO_2, mmHg	39(33,48)	40(34,50)	0.058
PH	7.37(7.3,7.4)	7.34(7.2,7.4)	<0.001
Lactate,mmol/L	2.2(1.6,3.3)	2.9(1.8,5.2)	<0.001
**Score**			
SAPSII	40(31,51)	53(42,63)	<0.001
SOFA	6(4,9)	9(7,11)	<0.001
**Treatment**			
FFP Trans, %	71(9.65)	67(17.72)	<0.001
RBC Trans, %	151(20.52)	94(24.87)	0.097
Dopamine, %	123(16.71)	96(25.40)	0.001
Epinephrine, %	82(11.14)	55(14.55)	0.101
Norepinephrine, %	214(29.08)	197(52.12)	<0.001
Phenylephrine, %	133(18.07)	87(23.02)	0.050
RRT, %	28(3.80)	47(12.43)	<0.001
MV, %	351(47.69)	253(66.93)	<0.001

SpO2: saturation of pulse oxygen; MCHC: Mean corpuscular hemoglobin concentration; MCV: Mean Corpuscular Volume; RBC: Red blood cell count; RDW: Red blood cell distribution width; WBC: white blood cell count; ALT: alanine transaminase; AST: aspartate transaminase; TBIL: total bilirubin; BUN: blood urea nitrogen; SCr: Serum creatinine; PT: Prothrombin time; INR: international normalized ratio; PaO_2: arterial partial pressure of oxygen; $PaCO_2$: arterial partial pressure of carbon dioxide; SOFA: sequential organ failure assessment; SAPS: simplified acute physiology score. FFP Trans: fresh frozen plasma Transfusion; RBC trans: Red Blood Cell Transfusion; RRT: Renal replacement therapy; MV: mechanical ventilation

Table S5: Feature ranking during recursive feature elimination

Feature	Rank	Feature	Rank
Age(years)	1	PO2(mmHg)	9
Gender(Female)	1	AST(IU/L)	10
Atrial fibrillation	1	Temperature(℃)	11
Systolic Pressure(mmHg)	1	Chloride(mmol/L)	12
SpO2(%)	1	PT(s)	13
Urine output(ml/24h)	1	Potassium(mmol/L)	14
Hemoglobin(g/dL)	1	Total Bilirubin(g/dL)	15
MCHC(g/dL)	1	Calcium(mmol/L)	16
Platelet(10^9/L)	1	Cerebrovascular disease	17
RDW(%)	1	Hypothyroidism	18
WBC(10^9/L)	1	PaCO2(mmHg)	19
Anion gap(mmol/L)	1	Dyslipidemia	20
Sodium(mmol/L)	1	Epinephrine	21
BUN(mg/dL)	1	RBC(10^12/L)	22
Creatinine(mg/dL)	1	Respiratory rate(bpm)	23
ALT(IU/L)	1	Peripheral vascular disease	24
Lactate(mmol/L)	1	Chronic kidney disease	25
RBC Trans	1	Coronary surgery history	26
Dopamine	1	RRT	27
Norepinephrine	1	Hypertension	28
MV	1	Old myocardial infarction	29
PH	2	Bicarbonate(mmol/L)	30
FFP Trans	3	Cancer	31
Hematocrit	4	MCV(fL)	32
Heart rate(bpm)	5	Diabetes	33
INR	6	Chronic pulmonary disease	34
Glucose(mg/dL)	7	Cardiomyopathy	35
Diastolic pressure(mmHg)	8	Phenylephrine	36

SpO2: saturation of pulse oxygen; MCHC: Mean corpuscular hemoglobin concentration; RDW: Red blood cell distribution width ; WBC: white blood cell count ; BUN: blood urea nitrogen; ALT: alanine transaminase; RBC trans: Red Blood Cell Transfusion; MV: mechanical ventilation; FFP Trans: fresh frozen plasma Transfusion; INR: international normalized ratio; PaO_2: arterial partial pressure of oxygen; AST: aspartate transaminase; PT: Prothrombin time; $PaCO_2$: arterial partial pressure of carbon dioxide; RBC: Red blood cell count; RRT: Renal replacement therapy; MCV: Mean Corpuscular Volume;

Table S6. Hyperparameter search domains and final settings of seven model

Hyperparameters	Type	Search domain	Final setting
**Logistic Regression**			
'C'	Float	(0.001,1)	0.565
'penalty'	categorical	['none', 'l2']	l2
'max_iter'	Int	[100,300]	275
**DecisionTree**			
'max_depth'	Int	[2,4]	4
'min_samples_split'	Int	[2,10]	2
'min_samples_leaf'	Int	[2,10]	6
'max_leaf_node'	Int	[20,40]	20
**SVM**			
'C'	Float	(0.001,200)	199.817981
'degree'	Int	[2,5]	4
**Random Forest**			
'max_depth'	Int	[2,3,4]	4
'n_estimators'	Int	[40,130]	104
'max_features'	Int	[5,20]	6
'min_samples_split'	Int	[2,15]	5
'min_samples_leaf'	Int	[2,10]	3
**CatBoost**			
'depth'	Int	[1,4]	4
'learning_rate'	Float	(0.001,0.004)	0.004
'bagging_temperature'	Float	(0,4)	3.0001
'reg_lambda'	Int	[1,5]	1
'min_data_in_leaf'	Int	[1,4]	3
**LightGBM**			
'max_depth'	Int	[1,2]	2
'learning_rate'	Float	(0.16,0.24)	0.2339
'n_estimators'	Int	[30,50]	49
'num_leaves'	Int	[30,50]	37
'subsample'	Float	(0.6,0.8)	0.53
**XGBoost**			
'max_depth'	Int	[1,2]	2
'learning_rate'	Float	(0.15,0.25)	0.239
'n_estimators'	Int	[30,50]	50
'min_child_weight'	Int	[4,15]	5
'subsample'	Float	(0.6,0.8)	0.6699

SVM: support vector machine; XGBoost: eXtreme Gradient Boosting; CatBoost: categorical boosting; LightGBM: light gradient boosting machine;

Table S7 Performance of the models in a 10-fold cross-validation

Model	Mean AUC(SD)	Mean Accuracy (SD)	Mean Recall (SD)	Mean F1-Score (SD)	Mean Brier Score (SD)
Logistic Regression	0.726(0.038)	0.673(0.027)	0.671(0.059)	0.671(0.036)	0.215(0.014)
Decision Tree	0.712(0.063)	0.687(0.049)	0.688(0.120)	0.687(0.065)	0.222(0.029)
SVM	0.710(0.040)	0.654(0.036)	0.644(0.050)	0.650(0.041)	0.219(0.012)
Random Forest	0.808(0.045)	0.724(0.055)	0.704(0.081)	0.717(0.064)	0.189(0.014)
CatBoost	0.817(0.052)	0.732(0.054)	0.724(0.083)	0.728(0.059)	0.180(0.019)
XGBoost	0.823(0.060)	0.747(0.052)	0.735(0.089)	0.742(0.058)	0.172(0.029)
LightGBM	0.812(0.053)	0.730(0.056)	0.722(0.083)	0.726(0.061)	0.178(0.024)

SVM: support vector machine; XGBoost: eXtreme Gradient Boosting;CatBoost: categorical boosting; LightGBM :light gradient boosting machine;

Table S8:Collinearity checks for continuous variables were performed for multivariate Cox analysis.

Variable	Tolerance	VIF
Age(years)	0.864	1.157
Systolic Pressure(mmHg)	0.972	1.029
SpO2(%)	0.962	1.039
Urine output(ml/24h)	0.848	1.18
Hemoglobin(g/dL)	0.75	1.334
MCHC(g/dL)	0.818	1.223
RDW(%)	0.75	1.333
WBC(10^9/L)	0.918	1.089
Anion gap(mmol/L)	0.477	2.099
BUN(mg/dL)	0.504	1.985
Creatinine(mg/dL)	0.518	1.931
Lactate(mmol/L)	0.626	1.596

VIF: Variance inflation factor; SPO2: saturation of pulse oxygen; MCHC: mean corpuscular hemoglobin concentration; RDW: red blood cell distribution width; WBC: white blood cell count; BUN: blood urea nitrogen.

Table S9 Delong test of ROC curve

**Internal validation**						
	LR	DT	SVM	Random Forest	Catboost	XGBoost
DT	0.0033					
SVM	0.0002	0.8141				
Random Forest	0.0549	0.0001	0.0001			
Catboost	0.0117	0.0001	0.0001	0.1194		
XGBoost	0.3348	0.0001	0.0002	0.3844	0.0895	
LightGBM	0.1405	0.0001	0.0001	0.7975	0.2385	0.4118
**External validation**						
	LR	DT	SVM	Random Forest	Catboost	XGBoost
DT	0.8623					
SVM	0.0564	0.1195				
Random Forest	0.0009	0.0037	0.1406			
Catboost	0.0001	0.0022	0.0726	0.4589		
XGBoost	0.0008	0.0065	0.213	0.8769	0.4706	
LightGBM	0.001	0.0053	0.1521	0.9433	0.5974	0.8103
**CatBoost vs. S-Catboost vs. SAPS II vs. SOFA in Internal validation**						
	CatBoost	S-Catboost	SAPS II			
S-Catboost	0.0053					
SAPS II	0.0001	0.0076				
SOFA	0.0001	0.0009	0.2799			

ROC: receiver operating characteristic curve; LR: logistic regression; SVM: Support Vector Machine; DT: decision tree; XGBoost: eXtreme Gradient BoostingCatBoost: Categorical Boosting; LightGBM: Light Gradient Boosting Machine; S-Catboost: simplified Catboost model.

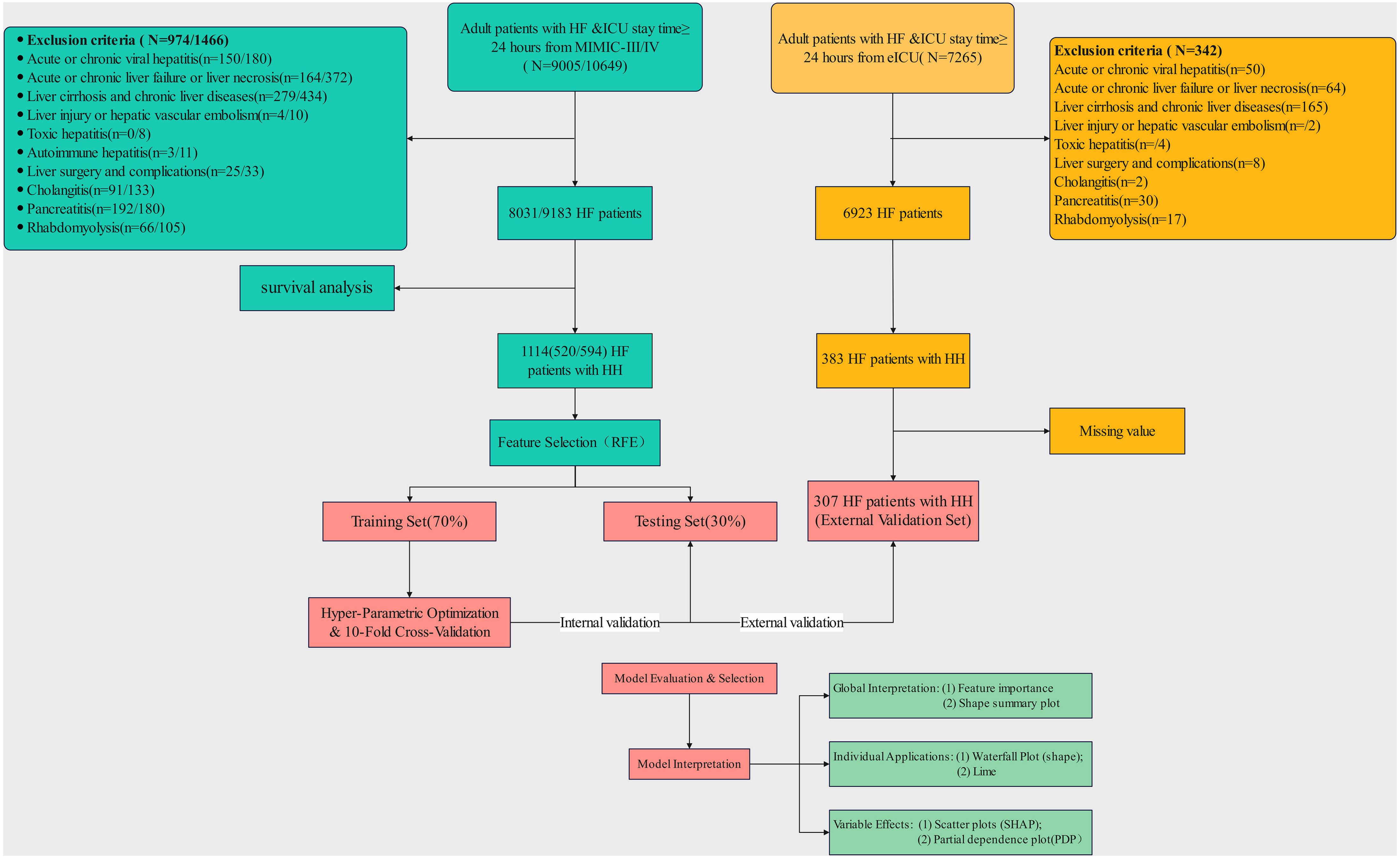

Figure S1: screening process of patients and the main flow of the study. MIMIC: Medical Information Mart for Intensive Care; EICU: The EICU Collaborative Database; HH: hypoxic hepatitis; HF: heart failure; RFE: feature recurrence elimination; SHAP: Shapley Additive Explanations; LIME: Local Interpretable Model-agnostic Explanations; PDP: Partial Dependence Plot.

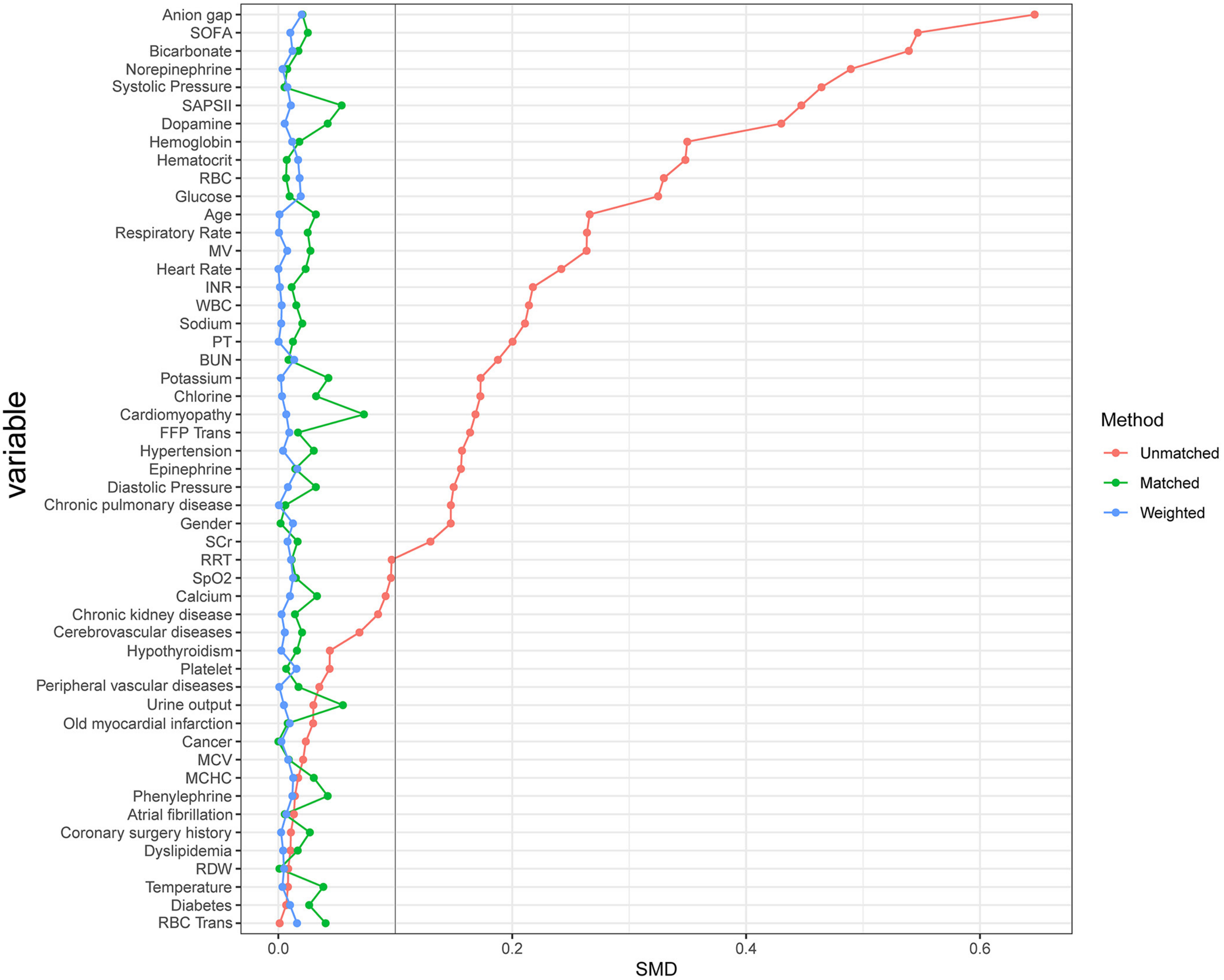

Figure S2: Standardized mean difference between the heart failure patients with and without hypoxic hepatitis before and after PSM**.** SMD: standardized mean difference; SpO2: saturation of pulse oxygen; MCHC: Mean corpuscular hemoglobin concentration; MCV: Mean Corpuscular Volume; RBC: Red blood cell count; RDW: Red blood cell distribution width; WBC: white blood cell count; BUN: blood urea nitrogen; SCr: Serum creatinine; PT: Prothrombin time; INR: international normalized ratio; SAPS II: The simplified acute physiology score II; SOFA: Sequential organ failure assessment; FFP Trans: fresh frozen plasma Transfusion; RBC trans: Red Blood Cell Transfusion; RRT: Renal replacement therapy; MV: mechanical ventilation

(A) Multivariate Cox Regression **(B)** Multivariate Cox Regression (PSM)

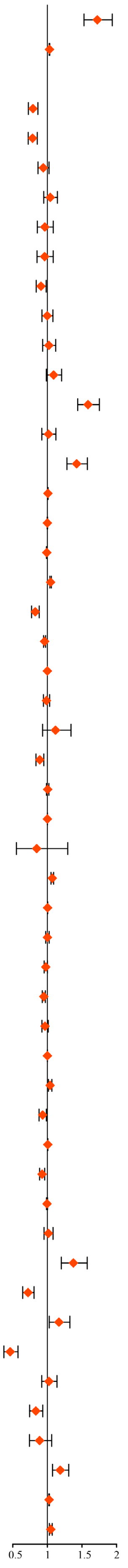

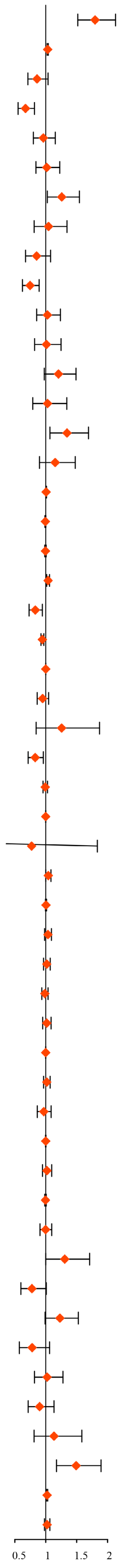

Variables	(A) HR [95%CI]	(A) p value	(B) HR [95%CI]	(B) p value
**Hypoxic hepatitis**	**1.722 (1.53−1.938)**	**<0.001**	**1.798 (1.518−2.128)**	**<0.001**
Age(years)	1.03 (1.026−1.035)	<0.001	1.032 (1.022−1.041)	<0.001
Gender	0.864 (0.803−0.929)	0.635	0.859 (0.711−1.037)	0.114
Hypertension	0.791 (0.726−0.863)	<0.001	0.672 (0.553−0.818)	<0.001
Dyslipidemia	0.786 (0.723−0.854)	<0.001	0.961 (0.8−1.154)	0.671
Diabetes	0.941 (0.865−1.024)	0.157	1.016 (0.842−1.227)	0.869
Coronary surgery history	1.042 (0.947−1.145)	0.401	1.259 (1.026−1.544)	0.028
Old myocardial infarction	0.963 (0.855−1.085)	0.536	1.045 (0.812−1.344)	0.733
Cardiomyopathy	0.96 (0.851−1.084)	0.514	0.852 (0.673−1.078)	0.182
Atrial fibrillation	0.908 (0.839−0.983)	0.017	0.746 (0.623−0.893)	0.001
Chronic pulmonary disease	0.998 (0.922−1.08)	0.956	1.027 (0.853−1.237)	0.777
Chronic kidney disease	1.022 (0.932−1.12)	0.649	1.011 (0.82−1.247)	0.918
Peripheral vascular diseases	1.09 (0.985−1.206)	0.094	1.206 (0.976−1.489)	0.082
Cerebrovascular diseases	1.587 (1.438−1.752)	<0.001	1.029 (0.79−1.34)	0.831
Hypothyroidism	1.016 (0.92−1.123)	0.752	1.344 (1.068−1.691)	0.012
Cancer	1.423 (1.283−1.578)	<0.001	1.152 (0.899−1.477)	0.263
Heart Rate (bpm)	1.008 (1.005−1.011)	<0.001	1.006 (1−1.012)	0.044
Systolic Pressure (mmHg)	1 (0.997−1.003)	0.913	0.994 (0.986−1.001)	0.11
Diastolic Pressure (mmHg)	0.991 (0.987−0.996)	<0.001	0.995 (0.984−1.007)	0.431
Respiratory Rate (bpm)	1.047 (1.036−1.057)	<0.001	1.038 (1.015−1.061)	0.001
Temperature (°C)	0.825 (0.771−0.882)	<0.001	0.831 (0.731−0.945)	0.005
SpO2 (%)	0.96 (0.945−0.975)	<0.001	0.945 (0.923−0.969)	<0.001
Urine output (ml/24h)	1 (1−1)	<0.001	1 (1−1)	0.026
Hematocrit (%)	0.987 (0.943−1.032)	0.561	0.95 (0.861−1.047)	0.298
Hemoglobin (g/dL)	1.117 (0.931−1.341)	0.234	1.257 (0.845−1.87)	0.26
MCHC (g/dL)	0.89 (0.835−0.948)	<0.001	0.829 (0.716−0.961)	0.013
MCV (fL)	1.004 (0.987−1.022)	0.615	0.992 (0.956−1.029)	0.655
Platelet (× 10^9/L)	1 (1−1)	0.278	0.999 (0.999−1)	0.156
RBC (× 10^12/L)	0.846 (0.552−1.294)	0.44	0.771 (0.324−1.834)	0.557
RDW (%)	1.071 (1.052−1.09)	<0.001	1.041 (1−1.084)	0.051
WBC (× 10^9/L)	1.003 (1.001−1.005)	0.006	1.004 (0.995−1.013)	0.39
Anion gap (mmol/L)	1.001 (0.976−1.026)	0.944	1.036 (0.981−1.092)	0.202
Bicarbonate (mmol/L)	0.977 (0.954−1.001)	0.057	1.017 (0.964−1.072)	0.54
Chlorine (mmol/L)	0.948 (0.926−0.971)	<0.001	0.986 (0.936−1.037)	0.577
Calcium (mmol/L)	0.966 (0.921−1.012)	0.148	1.016 (0.95−1.086)	0.64
Glucose (mg/dL)	1 (1−1.001)	0.615	0.999 (0.998−1)	0.154
Sodium (mmol/L)	1.04 (1.015−1.065)	0.001	1.017 (0.965−1.071)	0.534
Potassium (mmol/L)	0.931 (0.88−0.986)	0.014	0.969 (0.863−1.087)	0.59
BUN (mg/dL)	1.006 (1.004−1.008)	<0.001	1 (0.996−1.004)	0.848
SCr (mg/dL)	0.924 (0.888−0.961)	<0.001	1.019 (0.947−1.096)	0.614
PT (s)	0.995 (0.987−1.003)	0.206	0.996 (0.984−1.008)	0.498
INR	1.015 (0.952−1.082)	0.653	0.998 (0.907−1.097)	0.959
FFP Trans	1.376 (1.202−1.576)	<0.001	1.307 (0.999−1.71)	0.05
RBC Trans	0.721 (0.643−0.807)	<0.001	0.777 (0.598−1.009)	0.058
Dopamine	1.167 (1.027−1.325)	0.017	1.229 (0.989−1.527)	0.062
Epinephrine	0.462 (0.371−0.576)	<0.001	0.78 (0.573−1.062)	0.114
Norepinephrine	1.023 (0.918−1.139)	0.682	1.022 (0.818−1.278)	0.846
Phenylephrine	0.833 (0.743−0.933)	0.002	0.901 (0.716−1.134)	0.373
RRT	0.887 (0.742−1.062)	0.193	1.133 (0.811−1.584)	0.463
MV	1.186 (1.075−1.308)	0.001	1.491 (1.174−1.894)	0.001
SAPSII	1.025 (1.021−1.029)	<0.001	1.022 (1.012−1.031)	<0.001
SOFA	1.049 (1.029−1.07)	<0.001	1.022 (0.98−1.067)	0.313

Figure S3-1: Multivariate Cox analysis of 30-day mortality in patients with heart failure. (A) Before PSM; (B) After PSM. SpO2: saturation of pulse oxygen; MCHC: Mean corpuscular hemoglobin concentration; MCV: Mean Corpuscular Volume; RBC: Red blood cell count; RDW: Red blood cell distribution width; WBC: white blood cell count; BUN: blood urea nitrogen; SCr: Serum creatinine; PT: Prothrombin time; INR: international normalized ratio; SAPS II: The simplified acute physiology score II; SOFA: Sequential organ failure assessment; FFP Trans: fresh frozen plasma Transfusion; RBC trans: Red Blood Cell Transfusion; RRT: Renal replacement therapy; MV: mechanical ventilation

(A) Multivariate Cox Regression

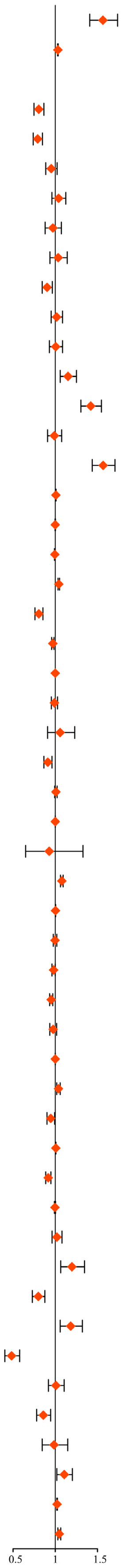

Variables	HR [95%CI]	p value
**Hypoxic hepatitis**	1.565 (1.408−1.738)	<0.001
Age (years)	1.031 (1.028−1.035)	<0.001
Gender	0.908 (0.854−0.966)	0.127
Hypertension	0.804 (0.748−0.865)	<0.001
Dyslipidemia	0.791 (0.739−0.848)	<0.001
Diabetes	0.952 (0.887−1.022)	0.175
Coronary surgery history	1.039 (0.96−1.125)	0.341
Old myocardial infarction	0.972 (0.88−1.072)	0.566
Cardiomyopathy	1.034 (0.937−1.142)	0.503
Atrial fibrillation	0.903 (0.845−0.965)	0.003
Chronic pulmonary disease	1.016 (0.952−1.086)	0.627
Chronic kidney disease	1.005 (0.93−1.086)	0.893
Peripheral vascular diseases	1.151 (1.059−1.251)	0.001
Cerebrovascular diseases	1.42 (1.304−1.547)	<0.001
Hypothyroidism	0.989 (0.909−1.076)	0.793
Cancer	1.567 (1.438−1.708)	<0.001
Heart Rate (bpm)	1.007 (1.005−1.01)	<0.001
Systolic Pressure (mmHg)	1 (0.997−1.002)	0.826
Diastolic Pressure (mmHg)	0.995 (0.991−0.999)	0.011
Respiratory Rate (bpm)	1.042 (1.033−1.051)	<0.001
Temperature (°C)	0.805 (0.759−0.854)	<0.001
SpO2 (%)	0.971 (0.957−0.985)	<0.001
Urine_output	1 (1−1)	<0.001
Hematocrit (%)	0.99 (0.954−1.027)	0.576
Hemoglobin (g/dL)	1.058 (0.909−1.231)	0.469
MCHC (g/dL)	0.912 (0.865−0.961)	0.001
MCV (fL)	1.008 (0.993−1.022)	0.304
Platelet (× 10^9/L)	1 (1−1)	0.483
RBC (× 10^12/L)	0.928 (0.649−1.328)	0.684
RDW (%)	1.077 (1.062−1.093)	<0.001
WBC (× 10^9/L)	1.003 (1.001−1.005)	0.009
Anion gap (mmol/L)	0.997 (0.977−1.018)	0.79
Bicarbonate (mmol/L)	0.98 (0.961−1)	0.049
Chlorine (mmol/L)	0.952 (0.933−0.971)	<0.001
Calcium (mmol/L)	0.975 (0.935−1.016)	0.23
Glucose (mg/dL)	1 (1−1)	0.822
Sodium (mmol/L)	1.038 (1.017−1.059)	<0.001
Potassium (mmol/L)	0.946 (0.902−0.993)	0.023
BUN (mg/dL)	1.006 (1.005−1.008)	<0.001
SCr (mg/dL)	0.917 (0.887−0.948)	<0.001
PT (s)	0.997 (0.99−1.004)	0.338
INR	1.019 (0.962−1.08)	0.519
FFP Trans	1.197 (1.064−1.347)	0.003
RBC Trans	0.799 (0.728−0.877)	<0.001
Dopamine	1.183 (1.059−1.321)	0.003
Epinephrine	0.482 (0.402−0.578)	<0.001
Norepinephrine	1.008 (0.919−1.105)	0.868
Phenylephrine	0.858 (0.779−0.945)	0.002
RRT	0.985 (0.846−1.148)	0.848
MV	1.107 (1.019−1.203)	0.017
SAPSII	1.022 (1.018−1.025)	<0.001
SOFA	1.048 (1.031−1.065)	<0.001

(B) Multivariate Cox Regression (PSM)

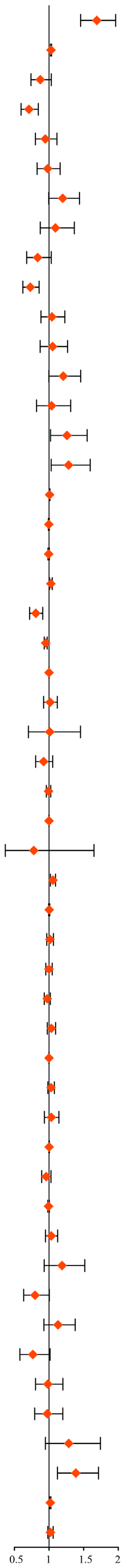

Variables	HR [95%CI]	p value
**Hypoxic hepatitis**	**1.69 (1.456−1.962)**	**<0.001**
Age (years)	1.029 (1.021−1.037)	<0.001
Gender	0.874 (0.739−1.033)	0.114
Hypertension	0.71 (0.596−0.845)	<0.001
Dyslipidemia	0.946 (0.803−1.114)	0.507
Diabetes	0.98 (0.828−1.16)	0.815
Coronary surgery history	1.197 (0.996−1.44)	0.056
Old myocardial infarction	1.092 (0.874−1.365)	0.439
Cardiomyopathy	0.836 (0.677−1.032)	0.096
Atrial fibrillation	0.73 (0.622−0.857)	<0.001
Chronic pulmonary disease	1.042 (0.883−1.228)	0.628
Chronic kidney disease	1.052 (0.872−1.268)	0.599
Peripheral vascular diseases	1.206 (0.998−1.457)	0.053
Cerebrovascular diseases	1.037 (0.82−1.313)	0.761
Hypothyroidism	1.259 (1.022−1.551)	0.031
Cancer	1.282 (1.031−1.595)	0.025
Heart Rate (bpm)	1.008 (1.003−1.014)	0.003
Systolic Pressure (mmHg)	0.997 (0.99−1.004)	0.386
Diastolic Pressure (mmHg)	0.995 (0.985−1.005)	0.304
Respiratory Rate (bpm)	1.026 (1.006−1.047)	0.01
Temperature (°C)	0.809 (0.72−0.909)	<0.001
SpO2 (%)	0.952 (0.931−0.974)	<0.001
Urine_output	1 (1−1)	0.001
Hematocrit (%)	1.016 (0.922−1.119)	0.754
Hemoglobin (g/dL)	1.011 (0.703−1.454)	0.954
MCHC (g/dL)	0.922 (0.807−1.053)	0.229
MCV (fL)	0.993 (0.961−1.025)	0.642
Platelet (× 10^9/L)	1 (0.999−1)	0.375
RBC (× 10^12/L)	0.78 (0.368−1.649)	0.515
RDW (%)	1.056 (1.02−1.094)	0.002
WBC (× 10^9/L)	1.002 (0.994−1.011)	0.57
Anion gap (mmol/L)	1.014 (0.967−1.064)	0.558
Bicarbonate (mmol/L)	0.998 (0.953−1.046)	0.948
Chlorine (mmol/L)	0.974 (0.931−1.019)	0.252
Calcium (mmol/L)	1.033 (0.975−1.095)	0.267
Glucose (mg/dL)	1 (0.999−1.001)	0.541
Sodium (mmol/L)	1.029 (0.983−1.077)	0.223
Potassium (mmol/L)	1.033 (0.933−1.143)	0.536
BUN (mg/dL)	1.002 (0.998−1.005)	0.329
SCr (mg/dL)	0.959 (0.894−1.028)	0.238
PT (s)	0.993 (0.982−1.005)	0.242
INR	1.033 (0.949−1.124)	0.457
FFP Trans	1.188 (0.93−1.516)	0.168
RBC Trans	0.798 (0.634−1.004)	0.054
Dopamine	1.13 (0.926−1.378)	0.229
Epinephrine	0.766 (0.579−1.015)	0.063
Norepinephrine	0.983 (0.805−1.2)	0.867
Phenylephrine	0.975 (0.793−1.199)	0.81
RRT	1.285 (0.947−1.742)	0.107
MV	1.387 (1.122−1.714)	0.003
SAPSII	1.019 (1.01−1.027)	<0.001
SOFA	1.02 (0.982−1.06)	0.305

Figure S3-2: Multivariate Cox analysis of 90-day mortality in patients with heart failure. (A) Before PSM; (B) After PSM. SpO2: saturation of pulse oxygen; MCHC: Mean corpuscular hemoglobin concentration; MCV: Mean Corpuscular Volume; RBC: Red blood cell count; RDW: Red blood cell distribution width; WBC: white blood cell count; BUN: blood urea nitrogen; SCr: Serum creatinine; PT: Prothrombin time; INR: international normalized ratio; SAPS II: The simplified acute physiology score II; SOFA: Sequential organ failure assessment; FFP Trans: fresh frozen plasma Transfusion; RBC trans: Red Blood Cell Transfusion; RRT: Renal replacement therapy; MV: mechanical ventilation

(A) Multivariate Cox Regression **(B)** Multivariate Cox Regression(PSM)

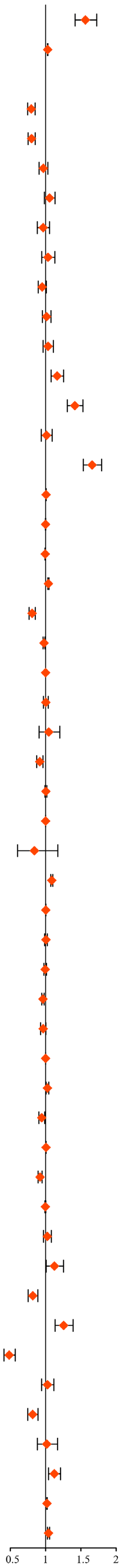

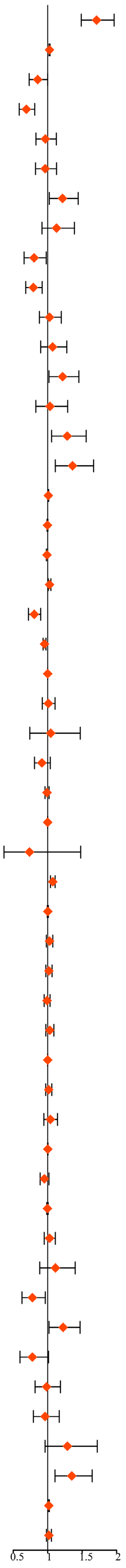

Variables	(A) HR [95%CI]	(A) p value	(B) HR [95%CI]	(B) p value
**Hypoxic hepatitis**	**1.564 (1.418−1.725)**	**<0.001**	**1.709 (1.487−1.964)**	**<0.001**
Age(years)	1.03 (1.026−1.033)	<0.001	1.026 (1.019−1.034)	<0.001
Gender	0.906 (0.857−0.958)	0.174	0.855 (0.731−1)	0.05
Hypertension	0.797 (0.746−0.852)	<0.001	0.69 (0.587−0.812)	<0.001
Dyslipidemia	0.801 (0.752−0.853)	<0.001	0.966 (0.83−1.125)	0.659
Diabetes	0.968 (0.908−1.032)	0.319	0.964 (0.823−1.128)	0.646
Coronary surgery history	1.056 (0.983−1.134)	0.139	1.215 (1.023−1.442)	0.026
Old myocardial infarction	0.965 (0.882−1.056)	0.441	1.128 (0.917−1.388)	0.254
Cardiomyopathy	1.034 (0.945−1.131)	0.467	0.802 (0.657−0.979)	0.03
Atrial fibrillation	0.951 (0.896−1.01)	0.105	0.791 (0.681−0.92)	0.002
Chronic pulmonary disease	1.014 (0.955−1.076)	0.658	1.026 (0.88−1.197)	0.74
Chronic kidney disease	1.035 (0.964−1.11)	0.343	1.071 (0.898−1.277)	0.444
Peripheral vascular diseases	1.163 (1.078−1.255)	<0.001	1.216 (1.018−1.452)	0.031
Cerebrovascular diseases	1.414 (1.308−1.529)	<0.001	1.033 (0.828−1.29)	0.772
Hypothyroidism	1.013 (0.939−1.093)	0.74	1.283 (1.056−1.558)	0.012
Cancer	1.659 (1.534−1.794)	<0.001	1.36 (1.111−1.666)	0.003
Heart Rate (bpm)	1.007 (1.004−1.009)	<0.001	1.009 (1.004−1.014)	<0.001
Systolic Pressure (mmHg)	1 (0.997−1.002)	0.669	0.997 (0.99−1.003)	0.315
Diastolic Pressure (mmHg)	0.995 (0.991−0.999)	0.007	0.99 (0.981−0.999)	0.035
Respiratory Rate (bpm)	1.04 (1.031−1.048)	<0.001	1.027 (1.008−1.046)	0.004
Temperature (℃)	0.809 (0.766−0.854)	<0.001	0.805 (0.721−0.898)	<0.001
SpO2 (%)	0.977 (0.964−0.991)	0.001	0.953 (0.933−0.975)	<0.001
Urine output (ml/24h)	1 (1−1)	<0.001	1 (1−1)	<0.001
Hematocrit (%)	1.003 (0.969−1.038)	0.864	1.01 (0.921−1.107)	0.833
Hemoglobin (g/dL)	1.044 (0.908−1.201)	0.544	1.044 (0.74−1.473)	0.805
MCHC (g/dL)	0.917 (0.874−0.963)	<0.001	0.916 (0.808−1.038)	0.167
MCV (fL)	1.004 (0.991−1.018)	0.549	0.99 (0.961−1.02)	0.508
Platelet (× 10^9/L)	1 (1−1)	0.881	1 (0.999−1)	0.213
RBC (× 10^12/L)	0.841 (0.603−1.173)	0.308	0.735 (0.366−1.479)	0.388
RDW (%)	1.087 (1.073−1.102)	<0.001	1.073 (1.04−1.108)	<0.001
WBC (× 10^9/L)	1.002 (1−1.004)	0.024	1.002 (0.994−1.01)	0.636
Anion gap (mmol/L)	1.005 (0.986−1.024)	0.605	1.026 (0.981−1.074)	0.263
Bicarbonate (mmol/L)	0.994 (0.976−1.012)	0.531	1.017 (0.973−1.063)	0.451
Chlorine (mmol/L)	0.963 (0.946−0.98)	<0.001	0.991 (0.949−1.034)	0.663
Calcium (mmol/L)	0.966 (0.929−1.004)	0.077	1.03 (0.973−1.09)	0.312
Glucose (mg/dL)	1 (1−1)	0.815	1 (0.999−1.001)	0.892
Sodium (mmol/L)	1.026 (1.007−1.045)	0.006	1.014 (0.971−1.06)	0.523
Potassium (mmol/L)	0.945 (0.904−0.987)	0.011	1.039 (0.945−1.142)	0.433
BUN (mg/dL)	1.006 (1.004−1.008)	<0.001	1.001 (0.998−1.005)	0.408
SCr (mg/dL)	0.921 (0.894−0.95)	<0.001	0.952 (0.89−1.018)	0.15
PT (s)	0.997 (0.99−1.003)	0.339	0.996 (0.986−1.007)	0.491
INR	1.024 (0.97−1.081)	0.393	1.027 (0.95−1.111)	0.504
FFP Trans	1.125 (1.008−1.254)	0.035	1.112 (0.884−1.399)	0.363
RBC Trans	0.819 (0.753−0.89)	<0.001	0.778 (0.627−0.965)	0.022
Dopamine	1.256 (1.135−1.39)	<0.001	1.224 (1.019−1.47)	0.031
Epinephrine	0.484 (0.41−0.57)	<0.001	0.778 (0.598−1.012)	0.061
Norepinephrine	1.026 (0.943−1.117)	0.545	0.984 (0.817−1.185)	0.864
Phenylephrine	0.817 (0.747−0.892)	<0.001	0.962 (0.792−1.168)	0.694
RRT	1.016 (0.883−1.17)	0.82	1.286 (0.962−1.719)	0.089
MV	1.123 (1.041−1.212)	0.003	1.349 (1.106−1.645)	0.003
SAPSII	1.019 (1.016−1.023)	<0.001	1.016 (1.008−1.024)	<0.001
SOFA	1.041 (1.026−1.057)	<0.001	1.017 (0.982−1.054)	0.342

Figure S3-3: Multivariate Cox analysis of 180-day mortality in patients with heart failure. (A) Before PSM; (B) After PSM. SpO2: saturation of pulse oxygen; MCHC: Mean corpuscular hemoglobin concentration; MCV: Mean Corpuscular Volume; RBC: Red blood cell count; RDW: Red blood cell distribution width; WBC: white blood cell count; BUN: blood urea nitrogen; SCr: Serum creatinine; PT: Prothrombin time; INR: international normalized ratio; SAPS II: The simplified acute physiology score II; SOFA: Sequential organ failure assessment; FFP Trans: fresh frozen plasma Transfusion; RBC trans: Red Blood Cell Transfusion; RRT: Renal replacement therapy; MV: mechanical ventilation

(A) Multivariate Cox Regression **(B)** Multivariate Cox Regression (PSM)

Variables	(A) HR [95%CI]	(A) p value	(B) HR [95%CI]	(B) p value
**Hypoxic hepatitis**	**1.45 (1.321–1.591)**	**<0.001**	**1.57 (1.379–1.788)**	**<0.001**
Age(years)	1.029 (1.026–1.032)	<0.001	1.025 (1.018–1.032)	<0.001
Gender	0.906 (0.862–0.953)	0.225	0.909 (0.785–1.054)	0.206
Hypertension	0.797 (0.75–0.847)	<0.001	0.702 (0.603–0.818)	<0.001
Dyslipidemia	0.798 (0.754–0.844)	<0.001	0.969 (0.84–1.118)	0.666
Diabetes	1.001 (0.945–1.06)	0.977	0.956 (0.824–1.109)	0.551
Coronary surgery history	1.032 (0.967–1.101)	0.343	1.172 (0.997–1.379)	0.055
Old myocardial infarction	0.945 (0.871–1.025)	0.173	1.106 (0.91–1.345)	0.311
Cardiomyopathy	1.025 (0.945–1.112)	0.546	0.814 (0.676–0.98)	0.03
Atrial fibrillation	0.955 (0.905–1.009)	0.099	0.852 (0.739–0.981)	0.026
Chronic pulmonary disease	1.037 (0.983–1.095)	0.183	1.056 (0.915–1.219)	0.455
Chronic kidney disease	1.049 (0.984–1.118)	0.143	1.1 (0.932–1.298)	0.258
Peripheral vascular diseases	1.194 (1.115–1.278)	<0.001	1.284 (1.089–1.515)	0.003
Cerebrovascular diseases	1.35 (1.256–1.451)	<0.001	1.016 (0.825–1.251)	0.882
Hypothyroidism	1.003 (0.936–1.075)	0.925	1.254 (1.044–1.505)	0.015
Cancer	1.754 (1.634–1.883)	<0.001	1.454 (1.202–1.757)	<0.001
Heart Rate (bpm)	1.005 (1.004–1.007)	<0.001	1.008 (1.003–1.012)	0.001
Systolic Pressure (mmHg)	1 (0.998–1.002)	0.885	0.998 (0.992–1.004)	0.548
Diastolic Pressure (mmHg)	0.997 (0.993–1)	0.053	0.994 (0.985–1.002)	0.155
Respiratory Rate (bpm)	1.033 (1.025–1.04)	<0.001	1.02 (1.003–1.038)	0.023
Temperature (°C)	0.81 (0.771–0.852)	<0.001	0.83 (0.748–0.921)	<0.001
SpO2 (%)	0.982 (0.97–0.995)	0.006	0.957 (0.936–0.977)	<0.001
Urine output (ml/24h)	1 (1–1)	<0.001	1 (1–1)	<0.001
Hematocrit (%)	1.008 (0.978–1.039)	0.611	1.011 (0.927–1.103)	0.798
Hemoglobin (g/dL)	1.025 (0.903–1.162)	0.704	1.053 (0.763–1.455)	0.752
MCHC (g/dL)	0.929 (0.89–0.97)	0.001	0.919 (0.818–1.033)	0.156
MCV (fL)	1.005 (0.993–1.017)	0.411	0.994 (0.967–1.022)	0.675
Platelet (× 10^9/L)	1 (1–1)	0.988	1 (0.999–1)	0.299
RBC (× 10^12/L)	0.851 (0.629–1.151)	0.294	0.721 (0.374–1.393)	0.331
RDW (%)	1.095 (1.082–1.108)	<0.001	1.08 (1.049–1.113)	<0.001
WBC (× 10^9/L)	1.002 (1–1.004)	0.036	1 (0.992–1.008)	0.96
Anion gap (mmol/L)	1.008 (0.991–1.026)	0.368	1.028 (0.985–1.072)	0.21
Bicarbonate (mmol/L)	0.999 (0.982–1.015)	0.87	1.018 (0.977–1.062)	0.386
Chlorine (mmol/L)	0.966 (0.95–0.981)	<0.001	0.994 (0.955–1.035)	0.782
Calcium (mmol/L)	0.972 (0.938–1.008)	0.122	1.032 (0.979–1.088)	0.243
Glucose (mg/dL)	1 (1–1)	0.836	1 (0.999–1.001)	0.678
Sodium (mmol/L)	1.022 (1.005–1.039)	0.009	1.008 (0.967–1.05)	0.718
Potassium (mmol/L)	0.96 (0.923–0.999)	0.043	1.023 (0.935–1.118)	0.622
BUN (mg/dL)	1.006 (1.004–1.007)	<0.001	1.001 (0.998–1.004)	0.683
SCr (mg/dL)	0.939 (0.914–0.964)	<0.001	0.985 (0.928–1.046)	0.626
PT (s)	0.997 (0.991–1.002)	0.255	0.996 (0.987–1.006)	0.448
INR	1.036 (0.985–1.089)	0.174	1.036 (0.962–1.116)	0.348
FFP Trans	1.069 (0.968–1.182)	0.187	1.064 (0.857–1.322)	0.572
RBC Trans	0.863 (0.801–0.931)	<0.001	0.827 (0.675–1.012)	0.066
Dopamine	1.233 (1.122–1.355)	<0.001	1.23 (1.034–1.464)	0.019
Epinephrine	0.482 (0.416–0.559)	<0.001	0.746 (0.58–0.96)	0.023
Norepinephrine	1.013 (0.937–1.095)	0.741	0.965 (0.809–1.15)	0.687
Phenylephrine	0.826 (0.762–0.895)	<0.001	1.011 (0.842–1.213)	0.908
RRT	1.021 (0.899–1.16)	0.746	1.26 (0.956–1.661)	0.1
MV	1.086 (1.014–1.163)	0.019	1.271 (1.055–1.53)	0.012
SAPSII	1.018 (1.015–1.021)	<0.001	1.016 (1.008–1.023)	<0.001
SOFA	1.039 (1.025–1.053)	<0.001	1.016 (0.982–1.05)	0.356

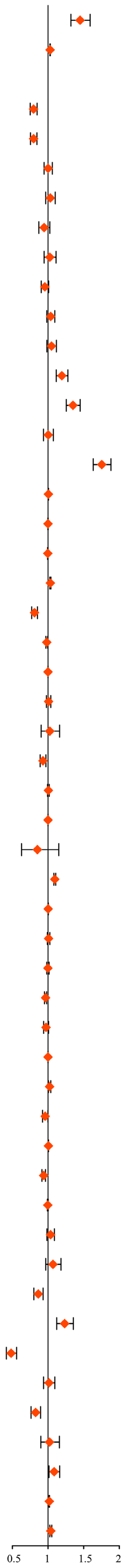

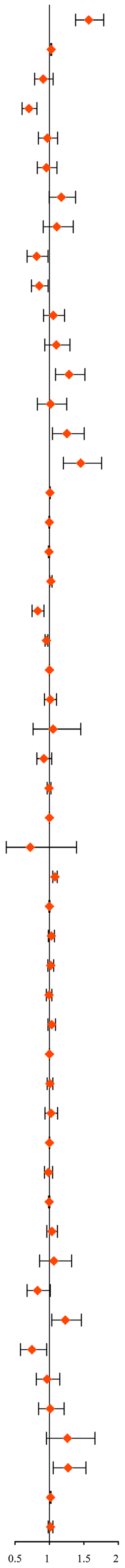

Figure S3-4: Multivariate Cox analysis of 365-day mortality in patients with heart failure. (A) Before PSM; (B) After PSM. SpO2: saturation of pulse oxygen; MCHC: Mean corpuscular hemoglobin concentration; MCV: Mean Corpuscular Volume; RBC: Red blood cell count; RDW: Red blood cell distribution width; WBC: white blood cell count; BUN: blood urea nitrogen; SCr: Serum creatinine; PT: Prothrombin time; INR: international normalized ratio; SAPS II: The simplified acute physiology score II; SOFA: Sequential organ failure assessment; FFP Trans: fresh frozen plasma Transfusion; RBC trans: Red Blood Cell Transfusion; RRT: Renal replacement therapy; MV: mechanical ventilation

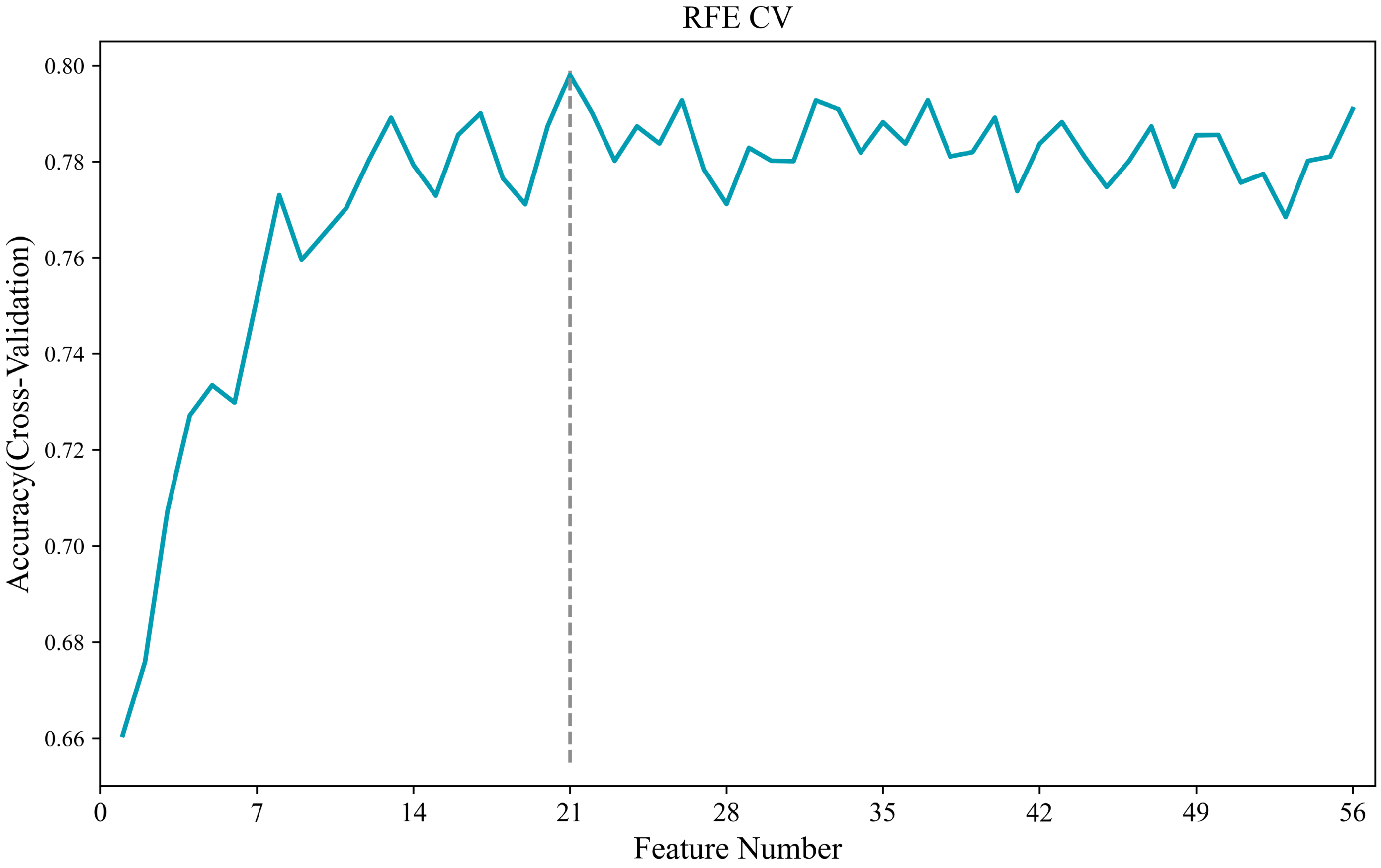

Figure S4: Number of features and corresponding accuracy scores in feature recursive elimination cross-validation (RFE-CV). The highest accuracy score was 0.798, achieved when the number of features was 21. The corresponding features were age, gender, atrial fibrillation, systolic Pressure, saturation of pulse oxygen (SPO2), urine output, hemoglobin, mean corpuscular hemoglobin concentration (MCHC), platelet, red blood cell distribution width (RDW), white blood cell count (WBC), anion gap, sodium, blood urea nitrogen (BUN), creatinine, alanine transaminase (ALT), Lactate, red blood cell transfusion (RBC Trans), dopamine, norepinephrine and mechanical ventilation (MV).

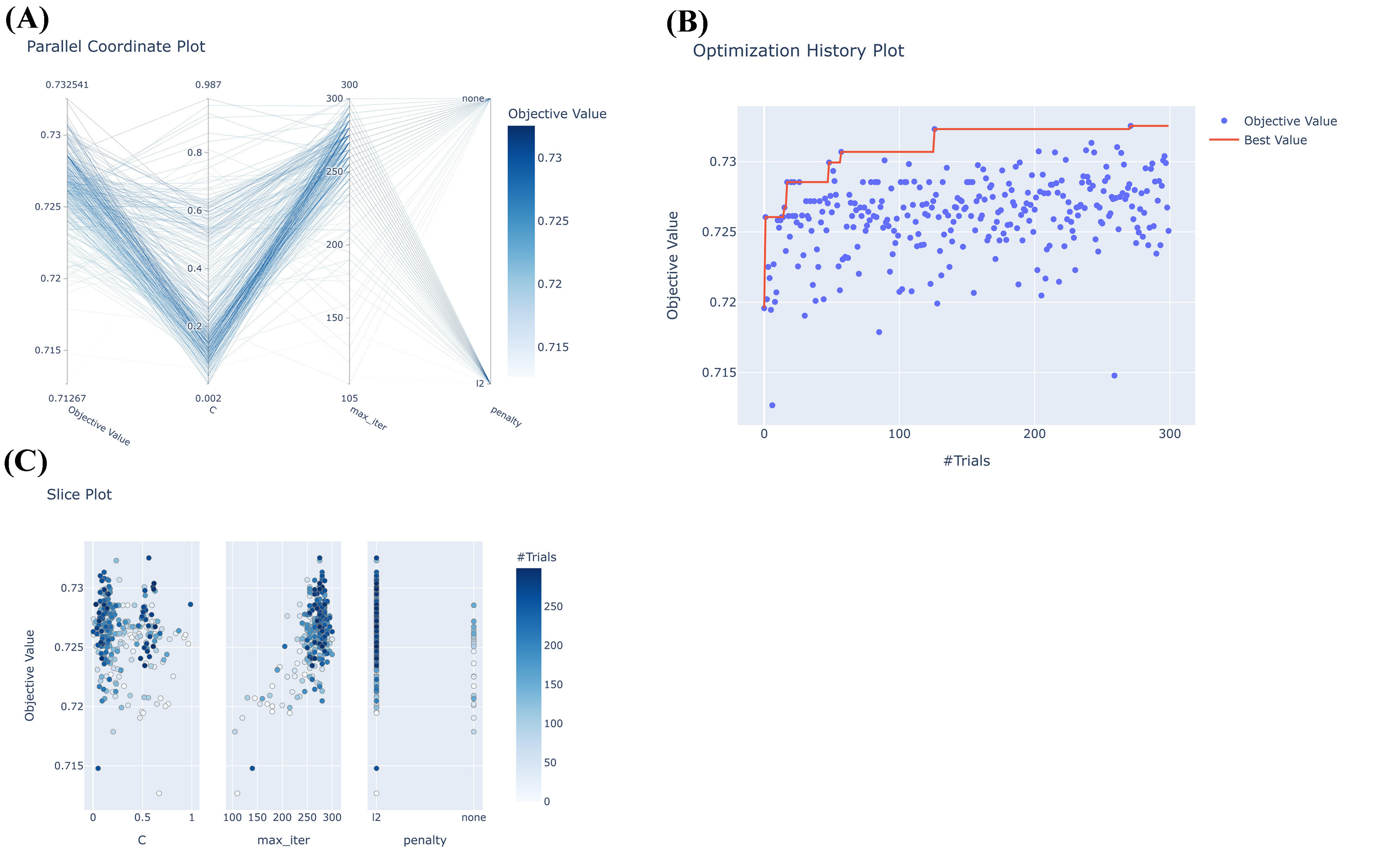

Figure5-1 Hyperparameter optimization process for the logistic regression model. (A) Parallel coordinate system plot of the hyperparametric distribution corresponding to different levels of AUC values, where darker colors correspond to greater AUC values; (B) Optimization history plot illustrating the evolution of optimal values during hyperparameter optimization; (C) Slice plot visualising the correlation between each parameter and the AUC.AUC: area under the curve

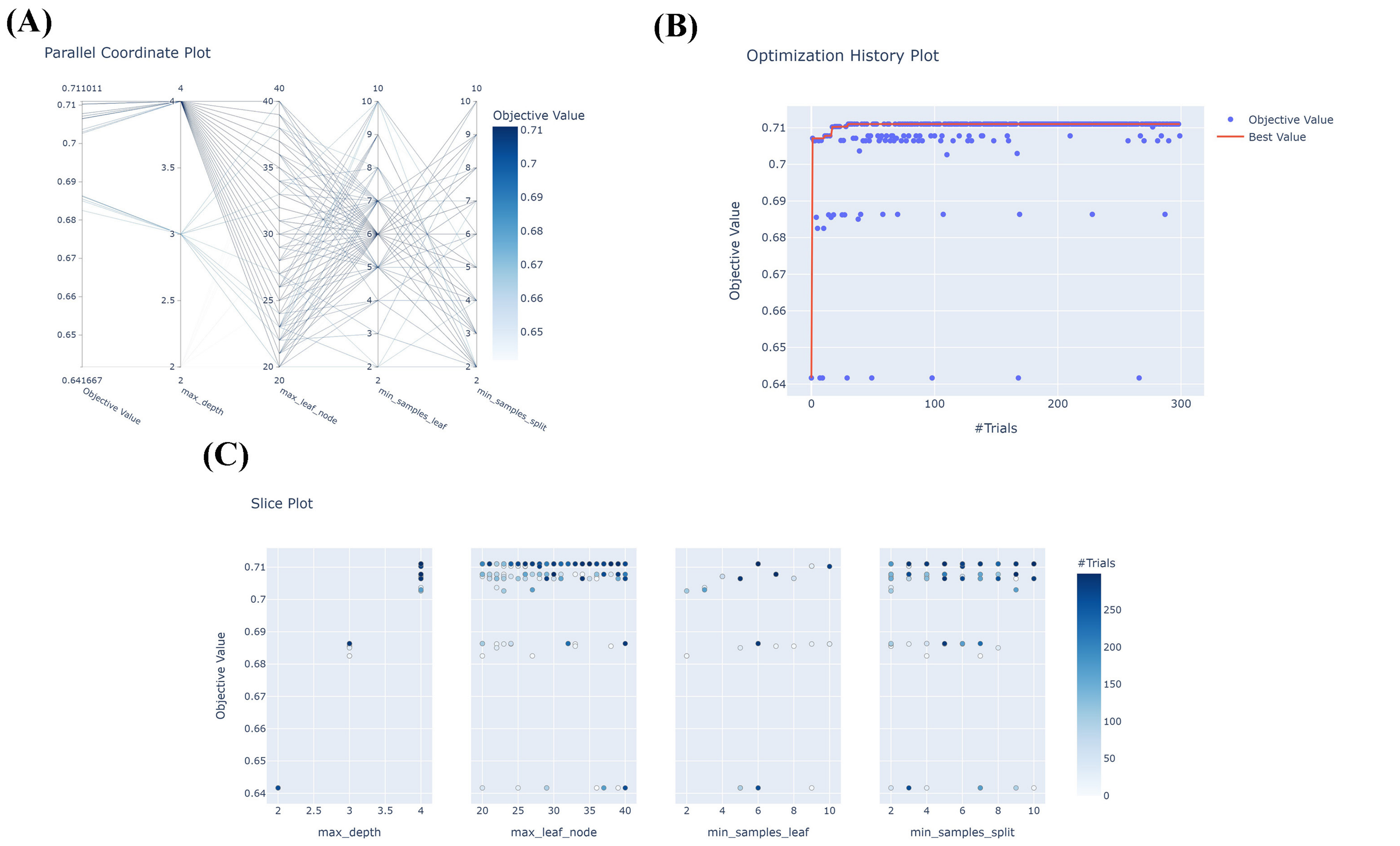

Figure5-2 Hyperparameter optimization process for the decision tree model. (A) Parallel coordinate system plot of the hyperparametric distribution corresponding to different levels of AUC values, where darker colors correspond to greater AUC values; (B) Optimization history plot illustrating the evolution of optimal values during hyperparameter optimization; (C) Slice plot visualising the correlation between each parameter and the AUC; AUC: area under the curve..

.

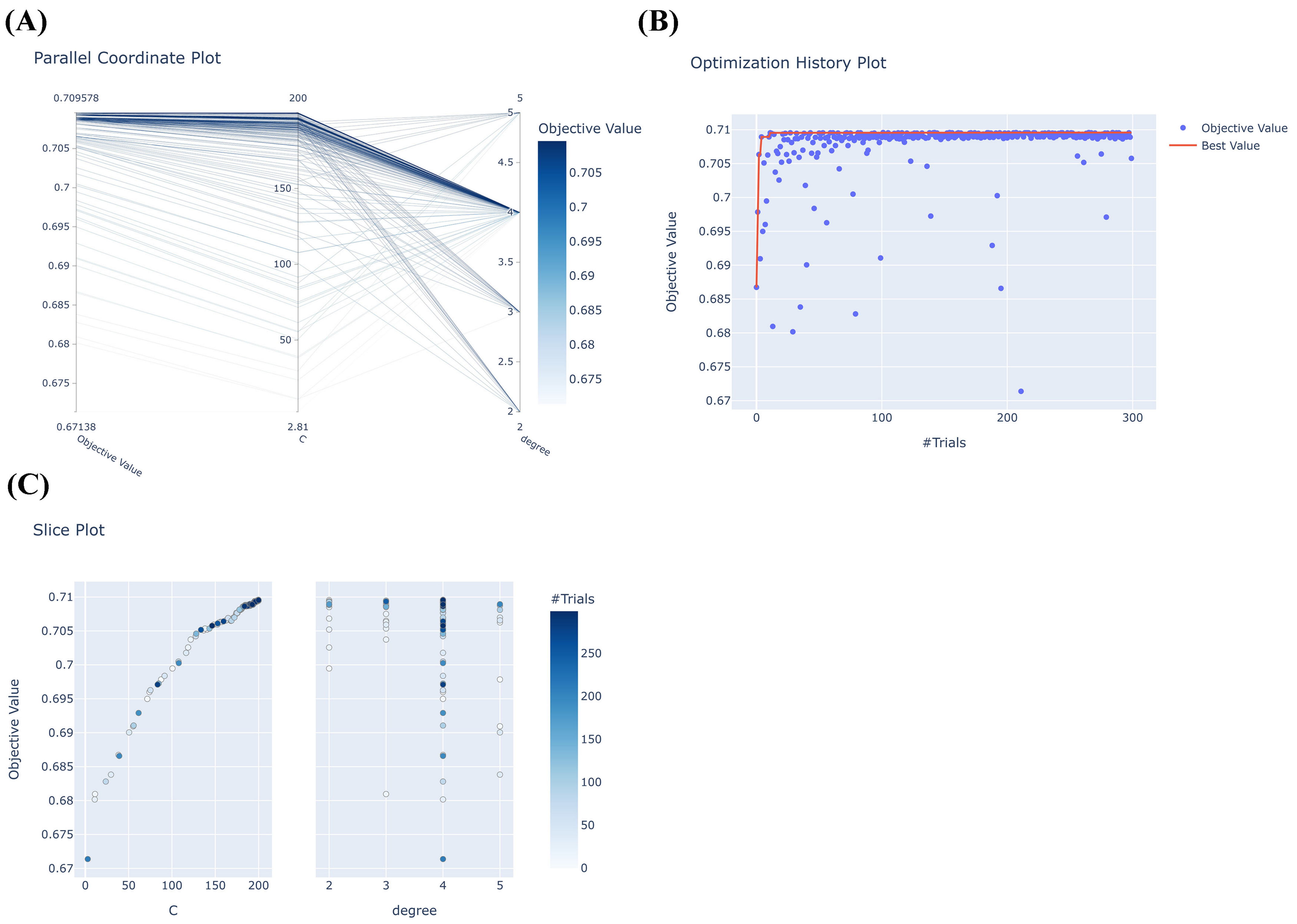

Figure5-3 Hyperparameter optimization process for the support vector machine model. (A) Parallel coordinate system plot of the hyperparametric distribution corresponding to different levels of AUC values, where darker colors correspond to greater AUC values; (B) Optimization history plot illustrating the evolution of optimal values during hyperparameter optimization; (C) Slice plot visualising the correlation between each parameter and the AUC.AUC: AUC: area under the curve.

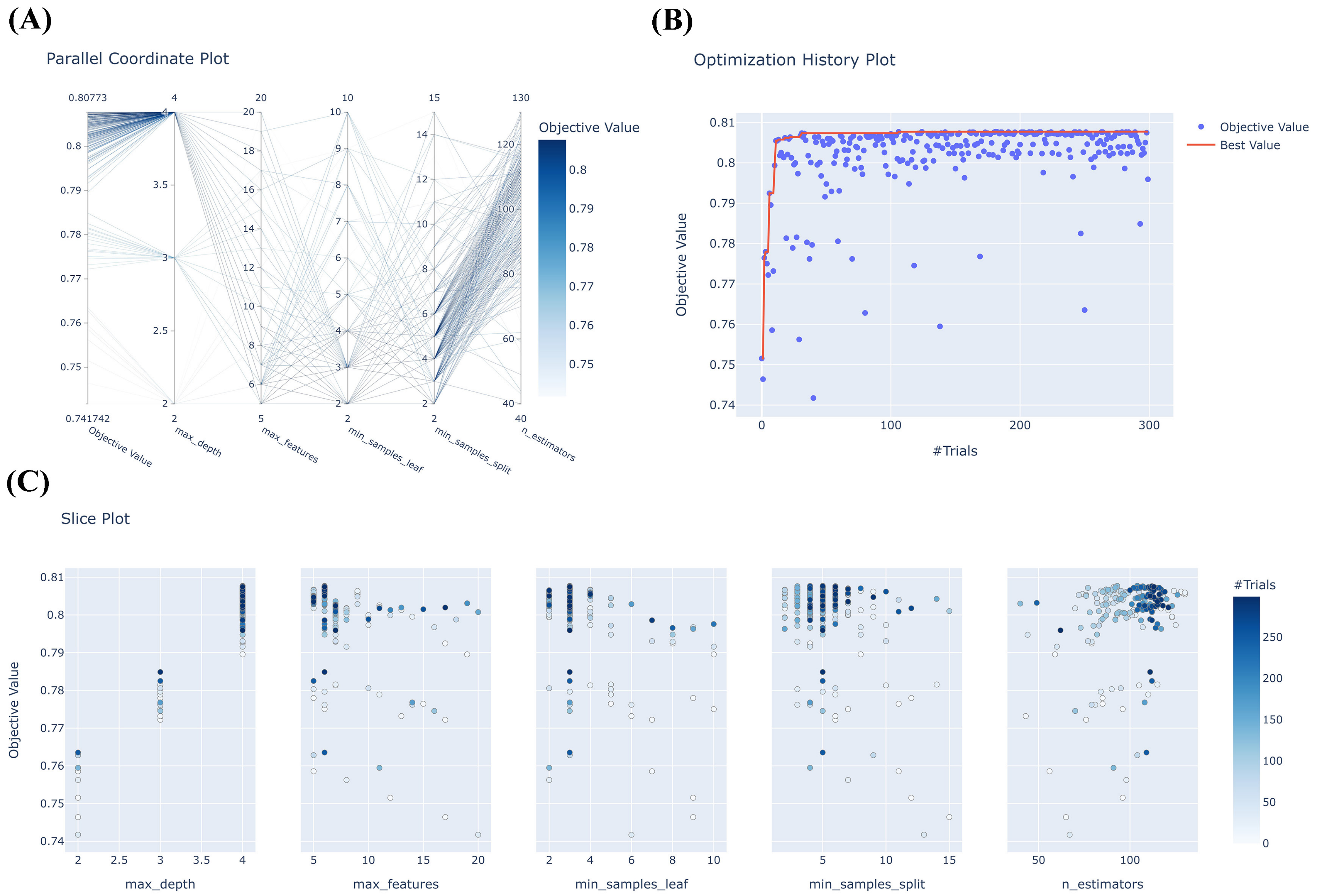

Figure5-4 Hyperparameter optimization process for the Random Forest model. (A) Parallel coordinate system plot of the hyperparametric distribution corresponding to different levels of AUC values, where darker colors correspond to greater AUC values; (B) Optimization history plot illustrating the evolution of optimal values during hyperparameter optimization; (C) Slice plot visualising the correlation between each parameter and the AUC.; AUC: area under the curve.

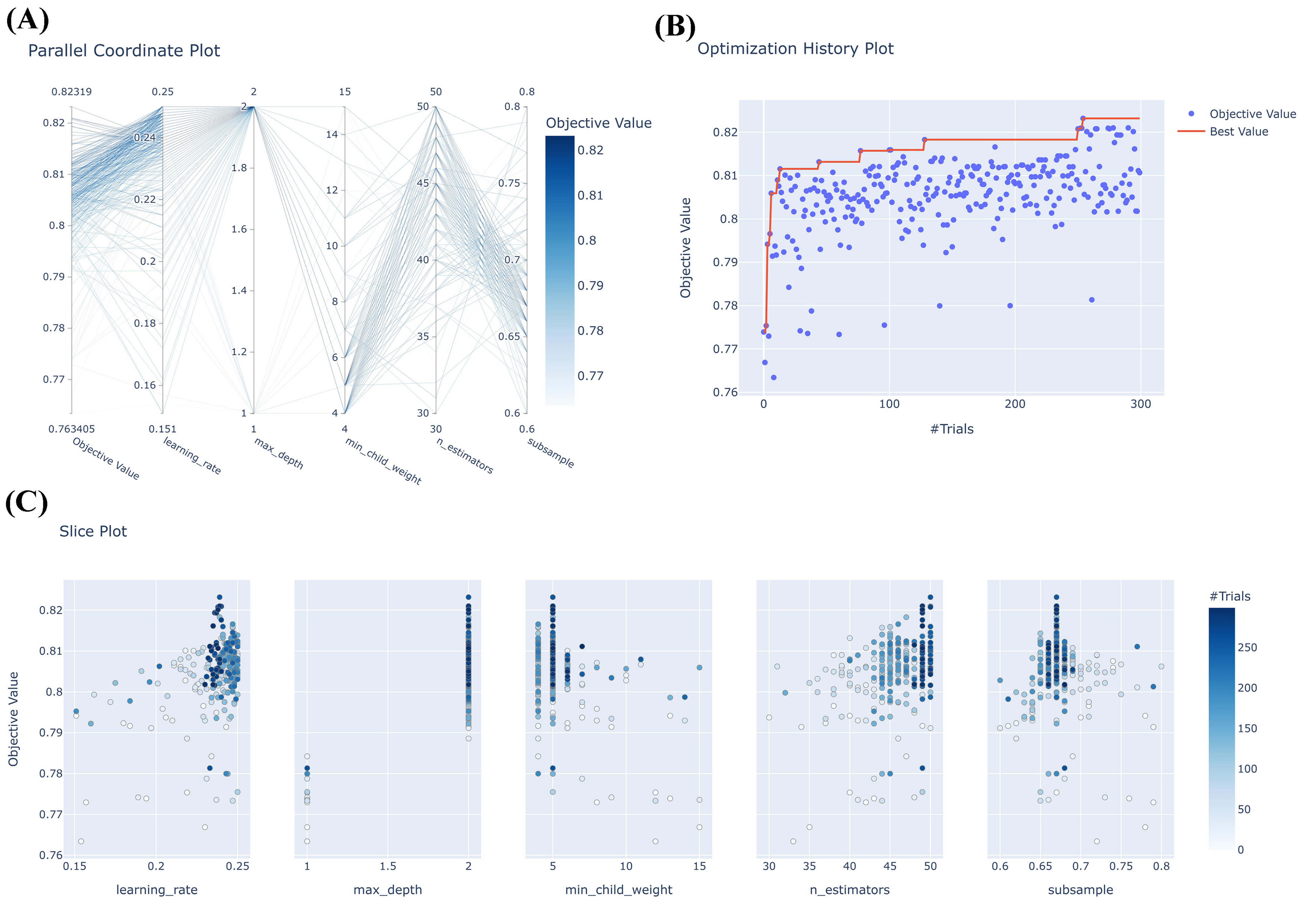

Figure5-5 Hyperparameter optimization process for the eXtreme Gradient Boosting model. (A) Parallel coordinate system plot of the hyperparametric distribution corresponding to different levels of AUC values, where darker colors correspond to greater AUC values; (B) Optimization history plot illustrating the evolution of optimal values during hyperparameter optimization; (C) Slice plot visualising the correlation between each parameter and the AUC.; AUC: area under the curve.

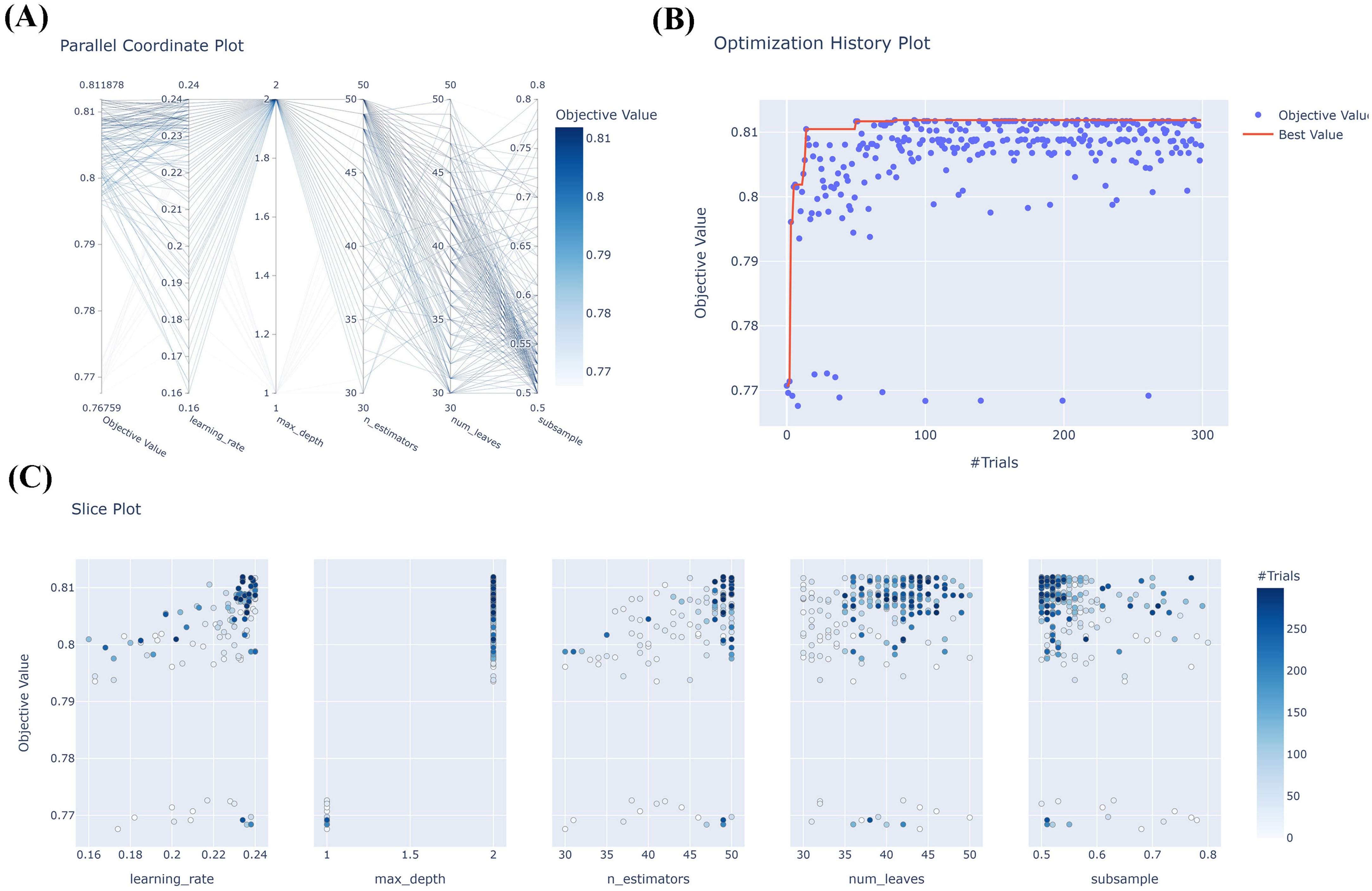

Figure5-6 Hyperparameter optimization process for the light gradient boosting machine model. (A) Parallel coordinate system plot of the hyperparametric distribution corresponding to different levels of AUC values, where darker colors correspond to greater AUC values; (B) Optimization history plot illustrating the evolution of optimal values during hyperparameter optimization; (C) Slice plot visualising the correlation between each parameter and the AUC; AUC: area under the curve.

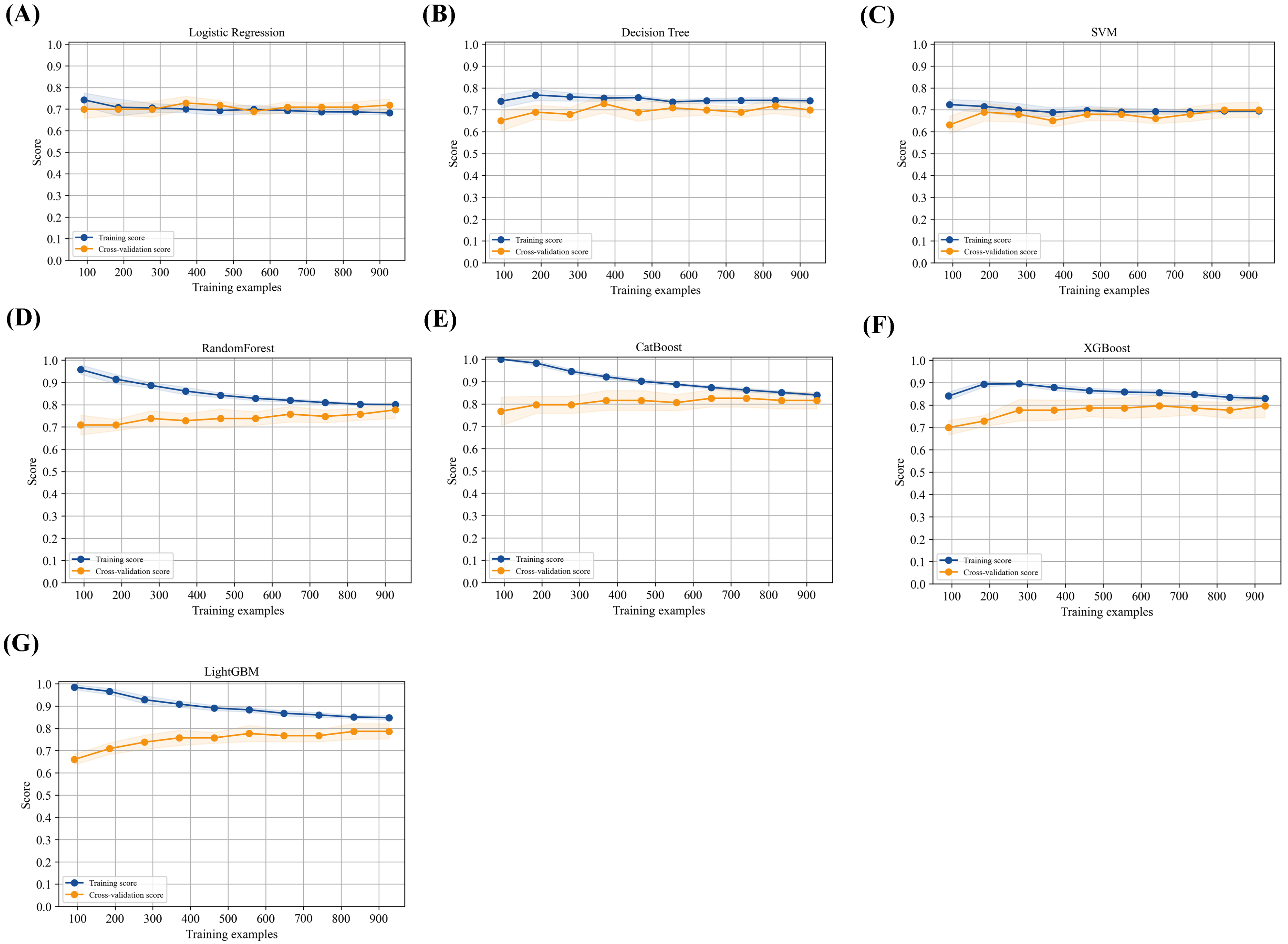

Figure S6 Learning curve of each model in the training process with final parameters. (A)Logistic Regression; (B) Decision Tree; (C)Support Vector Machine; (D)Random Forest; (E)Catboost; (F) XGBoost; (G)LightGBM. As the number of training samples increased, the error of the training set and test set decreased obviously and converged gradually. CatBoost: Categorical Boosting; XGBoost: eXtreme Gradient Boosting; LightGBM: Light Gradient Boosting Machine.

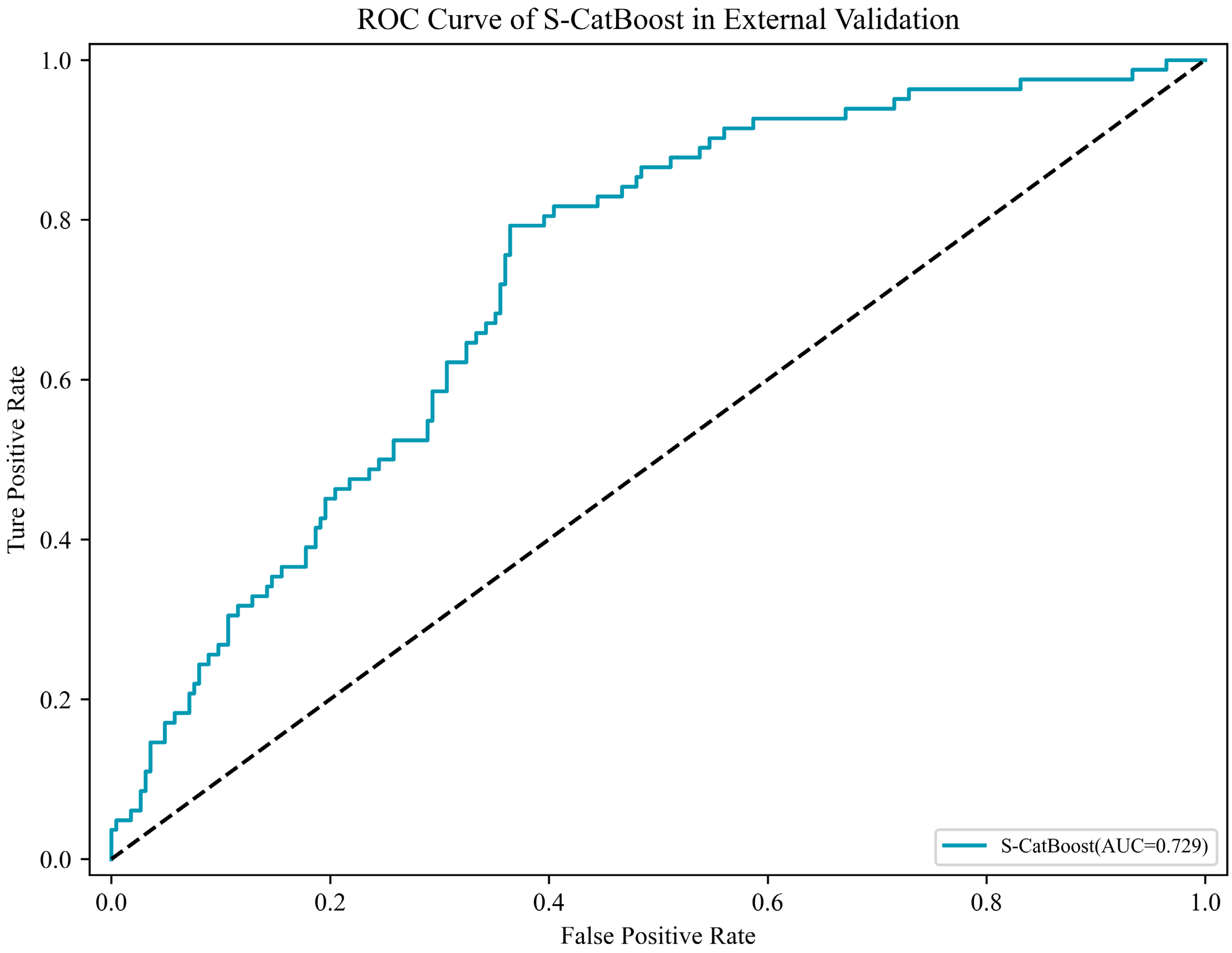

Figure S7 ROC curve of S-CatBoost in external validation. ROC: receiver operating characteristic curve; S-Catboost: simplified Catboost model. AUC: area under the curve.

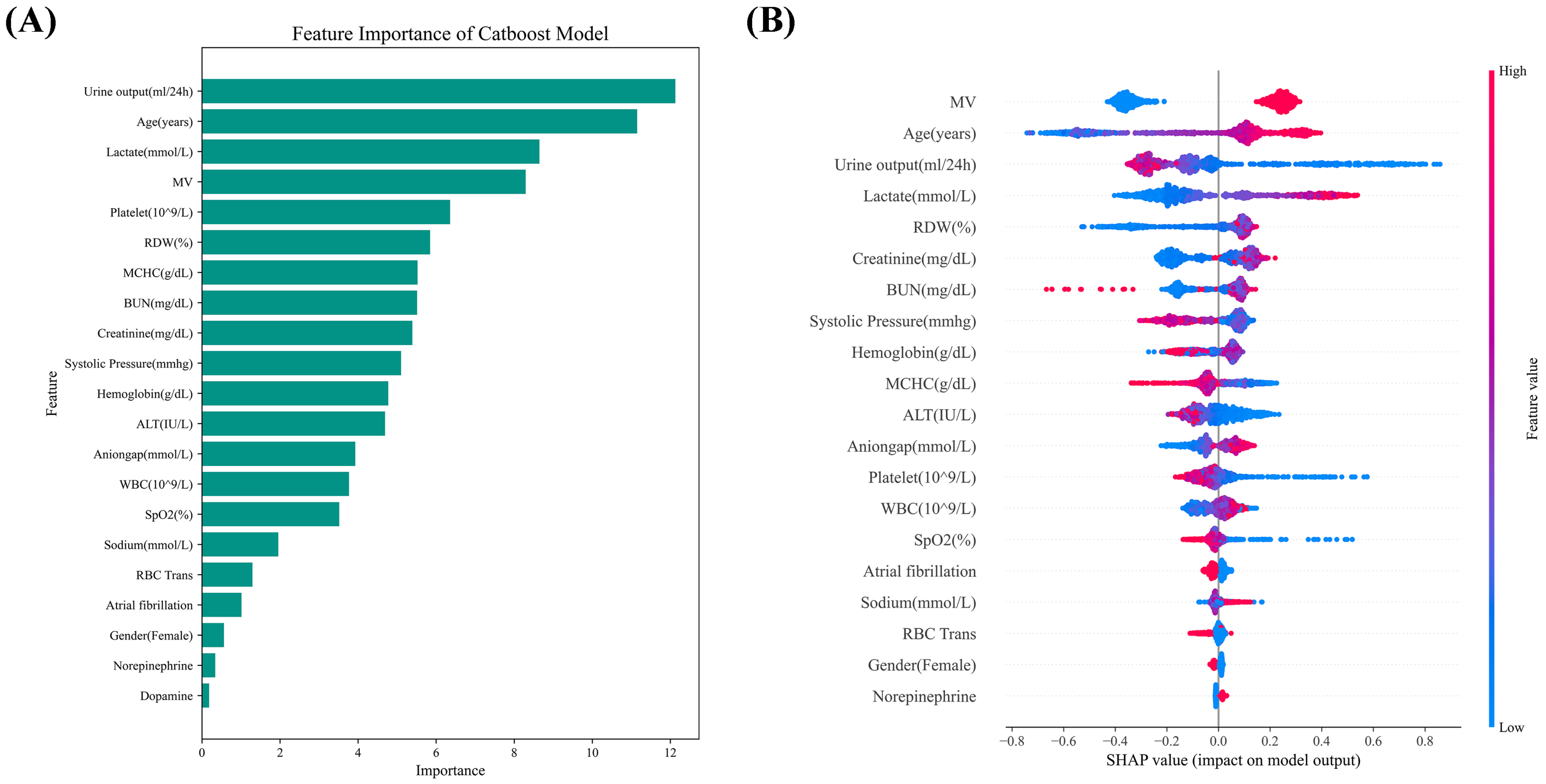

Figure S8: Feature importance ranking of the Catboost model. (A)Model importance ranking based on CatBoost;(B) Feature importance ranking based on the SHAP value. The vertical axis is from top to bottom, and the importance of features decreases. The position of the point on the horizontal axis indicates the feature's influence on the model's predicted value, and the point's color reflects the feature's value. For the numerical variable, the blue and red points represent lower and higher values; For categorical variables, blue and red dots correspond to yes or no, respectively. MV: mechanical ventilation; MCHC: mean corpuscular hemoglobin concentration; RDW: red blood cell distribution width; BUN: blood urea nitrogen; WBC: white blood cell; ALT: alanine transaminase; SPO2: saturation of pulse oxygen; RBC trans: Red Blood Cell Transfusion;

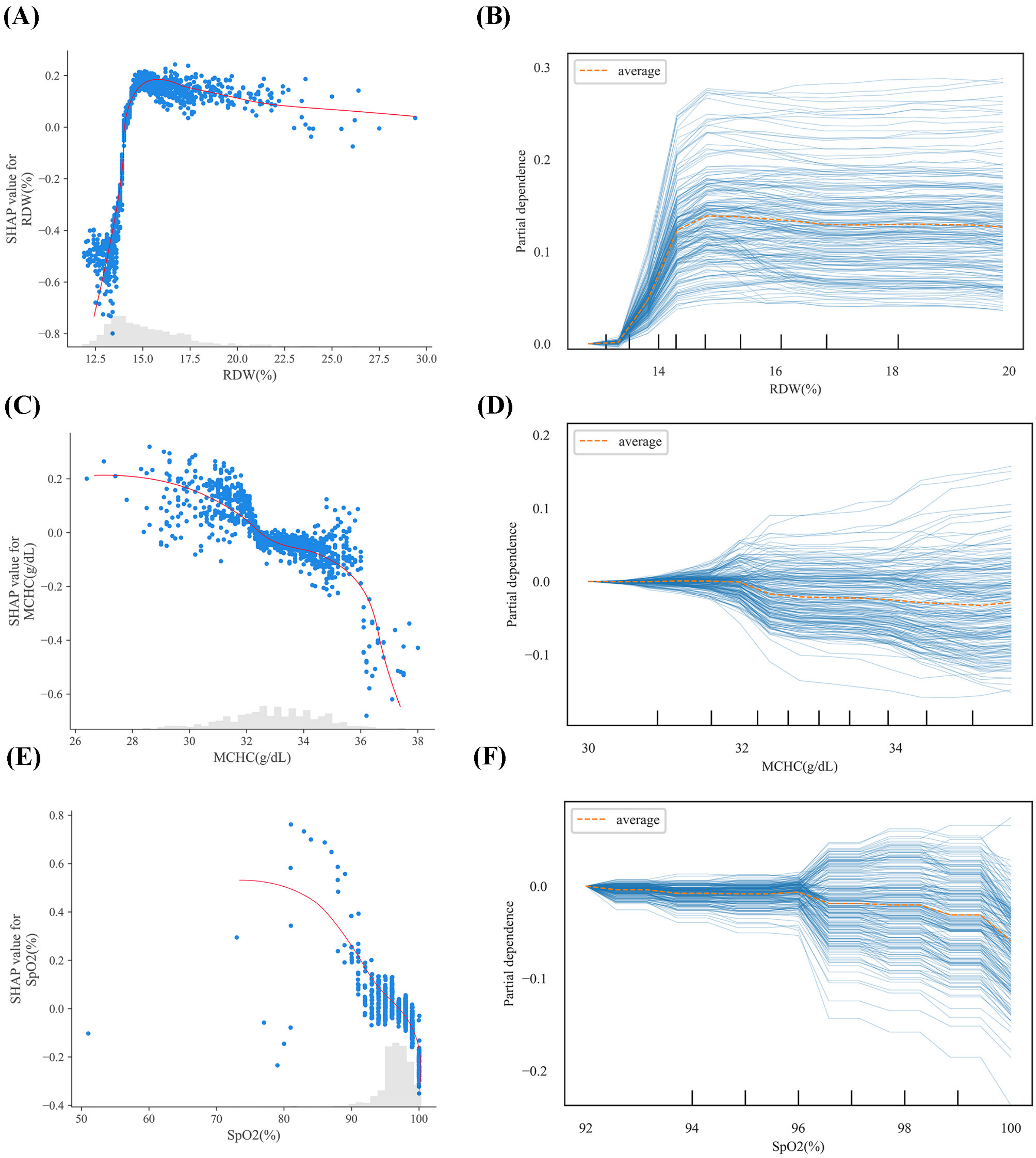

Fugure S9: Scatter plot (A,C,E) and PDP (B,D,F) of other continuous variables in S-CatBoost model.(A,B)RDW; (C,D) MCHC; (E,F) SPO2%.PDP:partial dependence plot; S-Catboost: simplified Catboost model;RDW: red blood cell distribution width;MCHC: mean corpuscular hemoglobin concentration;SPO2: saturation of pulse oxygen.
